# Tooth loss elevates all-cause and cause-specific mortality in adults with chronic kidney disease: The mediating role of frailty

**DOI:** 10.1097/MD.0000000000049843

**Published:** 2026-07-24

**Authors:** Jiaxin Shao, Yiren Bao, Kexin Zhang, Xinyi Zheng, Jiazhen Yin, Caifeng Zhu

**Affiliations:** a Hangzhou School of Clinical Medicine, Hangzhou Hospital of Traditional Chinese Medicine, Zhejiang Chinese Medical University, Hangzhou, China; b Department of Nephrology (Key Laboratory of Kidney Disease Prevention and Control Technology), Hangzhou Hospital of Traditional Chinese Medicine, Zhejiang Chinese Medical University, Hangzhou, China.

**Keywords:** chronic kidney disease, frailty, mortality, National Health and Nutrition Examination Survey (NHANES), oral health, tooth loss

## Abstract

Chronic kidney disease (CKD) patients experience a high oral disease burden and mortality. We evaluated whether loss of natural teeth predicts all-cause and cause-specific mortality in adults with CKD and whether a Frailty Index (FI) mediates this association. We analyzed 12,639 adults with CKD from the National Health and Nutrition Examination Survey 1999–2018. Tooth counts (third molars excluded) were grouped clinically. A 53-item FI was constructed. Survey-weighted Cox models with sensitivity checks, restricted cubic splines, piecewise Cox regression, and counterfactual mediation analysis were applied. Sequential models added FI and then ln(hs-CRP) to examine attenuation. Prespecified subgroup and sensitivity analyses were conducted, including cycles with prosthetic information. Greater tooth loss was independently associated with higher all-cause, cardiovascular, and cancer mortality. In fully adjusted models, compared with complete dentition, adjusted hazard ratios (HRs; 95% confidence intervals) for all-cause mortality were as follows: tooth loss, 1.53 (1.24–1.88); lacking functional, 1.93 (1.54–2.42); severe tooth loss, 1.99 (1.54–2.57); and edentulism, 2.16 (1.75–2.67). Spline analyses supported nonlinearity for all-cause, cardiovascular, and cancer mortality; segmented models were consistent with a steeper slope at lower tooth counts and a plateau thereafter. Piecewise analysis identified a threshold at 3 missing teeth: below the threshold, each additional missing tooth had an HR of 1.161 (1.108–1.216); above the threshold, HR was 1.010 (1.006–1.014) (log-rank *P* < .001). Associations were broadly consistent across sex and CKD risk strata, with relative effects attenuating at higher Kidney Disease: Improving Global Outcomes risk categories, where baseline hazards were greater. In cycles with prosthetic data, associations were weaker among participants receiving dental prosthetic rehabilitation. FI mediated 29.42% of the effect on all-cause mortality. Sequential adjustment indicated additional, smaller attenuation after ln(hs-CRP), while the primary associations remained significant. In US adults with CKD, tooth loss predicts higher mortality; frailty explains ~30% of this association, suggesting that the integration of routine oral health screening and dental interventions with frailty assessment and multidisciplinary care (nutritional support, rehabilitation, and psychosocial services) may identify at-risk CKD patients and offer modifiable targets to improve survival and quality of life.

## 1. Introduction

Chronic kidney disease (CKD), encompassing a spectrum of disorders affecting kidney structure and function, has an estimated global prevalence of 13.4%, representing a substantial worldwide public health burden.^[1,2]^ The US Centers for Disease Control and Prevention reports that CKD affects nearly 35.5 million US adults, corresponding to 14% of this population. Given its strong association with increased risks of death, cardiovascular disease (CVD), and progression to end-stage renal disease, CKD imposes a substantial economic and healthcare burden on both individuals and healthcare systems.^[3]^ Therefore, identifying underrecognized yet modifiable prognostic factors is crucial for improving outcomes in the CKD population.

Tooth loss, a prevalent oral health problem, serves as a critical marker for the accumulated impact of oral disorders.^[4]^ Findings from the National Health and Nutrition Examination Survey (NHANES) 2011 to 2016 reveal that in the cohort of adults aged ≥50 years, at least 59% exhibit loss of 8 or more teeth (excluding third molars).^[5]^ In the general population, tooth loss is correlated with malnutrition, systemic inflammation, dysbiosis, and an elevated risk of both all-cause and cardiovascular mortality.^[6]^ Moreover, it is also connected to greater susceptibility to chronic diseases, including CVD, diabetes, and dementia. Owing to metabolic disturbances, medication use, and immune dysfunction, CKD patients face heightened susceptibility to oral health problems, potentially worsening their systemic disease burden.^[7]^ Because tooth loss impairs mastication and dietary quality, timely dental prosthetic rehabilitation may partially restore function and nutrition and could attenuate downstream risk, although evidence in CKD-specific settings remains limited.

Tooth loss has also been increasingly recognized as a marker of frailty.^[8,9]^ By impairing mastication and nutritional intake, it accelerates muscle wasting and physical decline.^[6]^ In addition, oral inflammation associated with tooth loss contributes to cytokine imbalance, potentially impairing multisystem functions. Resulting facial changes and speech difficulties may lead to social withdrawal and depression,^[10]^ the latter being a major component of frailty. Previous studies have confirmed that frailty mediates mortality in the general population, as well as among individuals with cancer and multiple comorbidities.^[11]^ Within the CKD population, frailty is especially common, exceeding a prevalence of 60% in the dialysis cohort and consistently predicting adverse outcomes.^[6]^ Even in pre-dialysis CKD stages, approximately 29.6% of patients are classified as frail at baseline, rising to 58.5% after a 2-year observation period.^[12]^ Collectively, these observations substantiate the hypothesis that complex interrelationships exist among tooth loss, CKD, frailty, and mortality.

However, prior studies examining the relationship between tooth loss and mortality have mainly focused on the general population. It is not always appropriate to directly apply observations from the general population to the CKD population. For CKD patients – a group facing both high oral disease burden and elevated mortality risk – relevant evidence remains scarce. More importantly, frailty, as a common syndrome in CKD, may play a key mediating role in this association, yet this hypothesis has not been explored. Consequently, this study utilized nationally representative NHANES 1999–2018 data to assess the connection between tooth loss and all-cause/cause-specific mortality among adults with CKD. Additionally, we assessed whether the Frailty Index (FI) mediates the link between tooth loss and mortality.

## 2. Methods

### 2.1. Study population and data source

**Figure 1.**
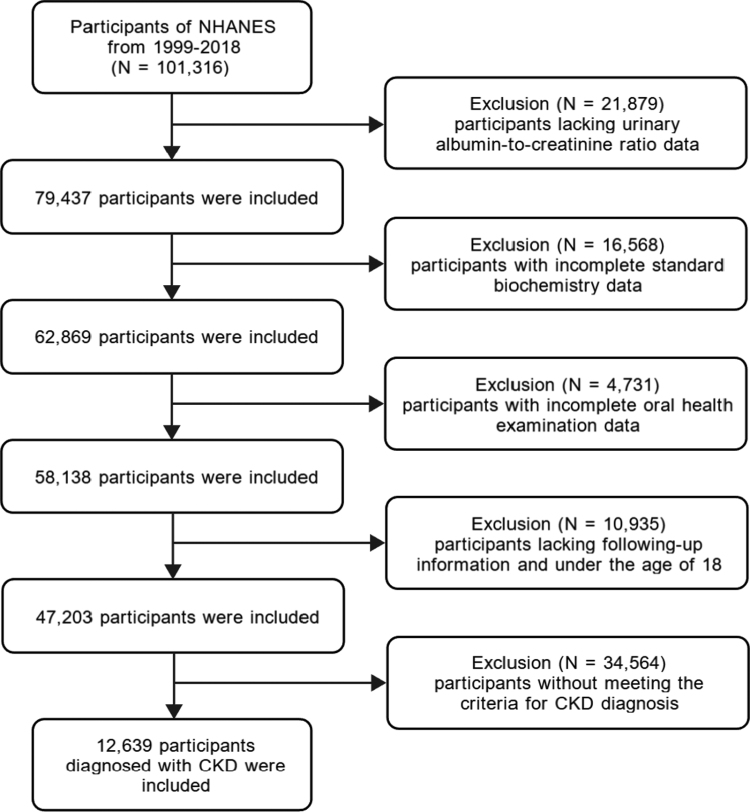
Flow diagram of study cohort selection. CKD = chronic kidney disease, NHANES = National Health and Nutrition Examination Survey.

This analysis utilized data from NHANES, conducted by the US Centers for Disease Control and Prevention and the National Center for Health Statistics (NCHS). The study complied with applicable Strengthening the Reporting of Observational Studies in Epidemiology guidelines for cohort studies and received approval from the NCHS Ethics Review Board. Signed written informed consent was obtained from all individuals. We applied the following exclusion criteria to the 101,316 participants in the 1999 to 2018 NHANES: 21,879 participants lacking urinary albumin-to-creatinine ratio (UACR) data; 16,568 participants with incomplete standard biochemistry profile datasets; 4731 participants with incomplete oral health examination (OHX) datasets; 10,935 participants excluded from mortality linkage files due to age <18 or incomplete follow-up data; and 34,564 participants without a CKD diagnosis based on standard criteria. The final cohort comprised 12,639 adults aged ≥18 years with confirmed CKD. Figure 1 illustrates the specific process of data selection.

### 2.2. Definition and risk stratification of CKD

CKD was characterized by either UACR ≥30 mg/g or estimated glomerular filtration rate (eGFR) <60 mL/min/1.73 m^2^. Although NHANES only provides single-time-point measurements related to eGFR and UACR, which do not meet the Kidney Disease: Improving Global Outcomes (KDIGO) criteria for kidney damage persisting for more than 3 months, the use of single measurements has been widely accepted as valid and effective in large-scale epidemiological studies.^[13]^ Since NHANES does not directly report eGFR, we calculated eGFR using the 2021 Chronic Kidney Disease Epidemiology Collaboration equation,^[14]^ expressed as mL/min/1.73 m^2^. Serum creatinine values were derived from the standard biochemistry profile dataset and measured using the kinetic Jaffe method. These values were calibrated to standardized creatinine levels from the Cleveland Clinic (Ohio) and expressed in mg/dL.

In the overall population, beyond defining CKD, we stratified risk using the KDIGO heat map that combines eGFR categories (G1–G5) with albuminuria (A1–A3). Participants with preserved filtration and normal albuminuria (eGFR ≥60 with UACR <30 mg/g) were treated as non-CKD/low risk. Moderate risk encompassed either mildly reduced filtration with normal albuminuria (G3a with A1) or preserved filtration with moderately increased albuminuria (G1–G2 with A2). High risk captured combinations indicating either a steeper reduction in filtration or heavier albuminuria without reaching the very highest strata (G3b with A1, G3a with A2, or G1–G2 with A3). Very high risk corresponded to severely reduced filtration regardless of albuminuria (G4–G5), as well as combinations where albuminuria and filtration jointly imply the highest risk (G3b with A2–A3 and G3a with A3).^[13]^

### 2.3. Tooth loss and dental prosthetic assessment

In the OHX dataset from NHANES, all 32 teeth were recorded individually as primary teeth, permanent teeth, dental implants, absent teeth, or permanent root fragments. Considering congenital agenesis of third molars and the common extraction of fully erupted third molars in early adulthood,^[15]^ third molars were excluded from the calculation of permanent tooth count. Natural tooth count was operationalized as the sum of permanent teeth present, excluding both root fragments and implants. Based on clinically meaningful cutoffs reported in previous studies, we categorized natural tooth count into 5 groups^[5]^: complete dentition (28 teeth), tooth loss (20–27 teeth), lacking functional (9–19 teeth), severe tooth loss (1–8 teeth), and edentulism (no natural teeth).

Dental prosthetic rehabilitation was ascertained from OHX prosthodontic items in cycles with available prosthetic information (1999–2004 and 2011–2018). We defined dental treatment (any vs none) and a 4-level category of dental treatment: removable denture only, fixed restoration/implant only, mixed restorations (both removable and fixed), and none. Because prosthetic items were not collected from 2005 to 2010, sensitivity analyses that explicitly incorporated prosthetic rehabilitation were restricted to cycles with available data, whereas the main analyses used the full 1999 to 2018 sample.

### 2.4. Definition of FI

The FI was calculated using the standard procedure proposed by Searle et al,^[16]^ which aggregates deficits across multiple physiological systems. In our study, a total of 53 deficits from 7 domains were incorporated, each assigned a score between 0 and 1 based on severity. The 7 domains were as follows: cognitive, 1 item regarding confusion or memory issues; dependency, 20 items assessing difficulty in activities of daily living; depression, 7 items derived from the Patient Health Questionnaire-9 assessing depressive symptoms; comorbidities, 13 self-reported chronic conditions, including arthritis, thyroid disorders, chronic bronchitis, malignancy, heart failure, coronary heart disease, angina, hypertension (HT), myocardial infarction, stroke, diabetes, kidney weakness, and urinary incontinence; healthcare utilization and access, 5 items regarding general health status, perceived health change over the past year, hospitalization, frequency of medical visits, and number of prescriptions; physical function and anthropometry, 1 item for body mass index (BMI); and laboratory measures, 6 items, including glycated hemoglobin, red blood cell count, hemoglobin, red cell distribution width, lymphocyte percentage, and segmented neutrophil percentage. FI was calculated as the ratio of the total number of deficits present to the total number assessed. The list of items and scoring criteria is provided in Table S1, Supplemental Digital Content 1.

### 2.5. Definition of high-sensitivity C-reactive protein

High-sensitivity C-reactive protein (hs-CRP) was measured at the NHANES central laboratory following standardized protocols described in the NHANES Laboratory Procedures Manuals. For analyses that explicitly included hs-CRP, we restricted to cycles with available hs-CRP data (1999–2010 and 2015–2018). To ensure comparability across assay and instrument changes, we applied the NCHS bridging/calibration guidance to place values on a common scale and express all concentrations in mg/L. Values below the limit of detection were set to LOD/√2. Given the potential for acute inflammatory states to bias associations, we conducted a sensitivity analysis excluding participants with hs-CRP >10 mg/L. For modeling, hs-CRP was natural-log transformed (ln[hs-CRP]).

### 2.6. Mortality, baseline, and follow-up

Mortality data for this study were obtained from the linked mortality files managed by the NCHS. NHANES participants were continuously linked to the National Death Index. The OHX date defined the commencement point. Person-months of follow-up were ascertained from this baseline until death, censoring, or December 31, 2019. Mortality outcomes included all-cause and cause-specific mortality. Given the established link between CKD and cardiovascular mortality, CVD mortality was defined by the International Classification of Diseases, 10th Revision (ICD-10) codes I00 to I09, I11, I13, I20 to I51. Cancer mortality was defined by ICD-10 codes C00 to C97, consistent with comparable International Classification of Diseases, Ninth Revision designations. Kidney disease mortality encompassed ICD-10 codes N00 to N07, N17 to N19, and N25 to N27, covering nephritis, nephrotic syndrome, and nephrosis.

### 2.7. Other covariates

Based on previous literature, we identified potential confounders of the association between tooth loss and mortality in CKD for inclusion in multivariable models. Covariates included demographic factors, lifestyle behaviors, clinical examinations, laboratory values, and comorbidity status: age (years), sex (self-reported male/female), race/ethnicity (Mexican American, Other Hispanic, non-Hispanic White, non-Hispanic Black, Other), marital status (married or in a relationship vs unmarried or single), education level (less than high school, high school or equivalent, college or above), BMI (kg/m^2^), smoking status (current, former, or never), poverty-income ratio, serum cotinine (COT, ng/mL), HT (self-report diagnosis, measured systolic blood pressure >140 mm Hg or diastolic blood pressure >90 mm Hg, or antihypertensive use), diabetes (American Diabetes Association’s criteria^[17]^: HbA1c ≥6.5%; 2-hour post-load plasma glucose ≥200 mg/dL; fasting glucose ≥125 mg/dL; self-reported diagnosis; or use of antidiabetic medication), CVD (self-reported congestive heart failure, coronary heart disease, angina, heart attack, or stroke), and hyperlipidemia (HPL; total cholesterol ≥200 mg/dL; triglycerides ≥150 mg/dL; high-density lipoprotein cholesterol <40 mg/dL in men or <50 mg/dL in women; or low-density lipoprotein cholesterol ≥130 mg/dL).

### 2.8. Statistical analysis

All statistical analyses accounted for the complex survey design of NHANES by applying the appropriate sampling weights and by considering the clustered and stratified sampling structure.^[18]^

Multiple imputation by chained equations was used for covariates with <10% missing data, in a manner tailored to survey data.^[19,20]^ Five multiply-imputed datasets were generated (with 1000 burn-in iterations and 1000 subsequent iterations) to ensure convergence of the imputation process.

Baseline characteristics were compared by mortality status. Continuous variables that approximately followed a normal distribution were presented as mean ± standard deviation, and categorical variables were summarized as counts and percentages (%). Group differences were evaluated using the Kruskal–Wallis test for continuous variables and a weighted chi-square test for categorical variables. In addition, overall survival by tooth-loss category was illustrated using Kaplan–Meier curves, and differences between these survival curves were compared with log-rank tests.

Cox proportional hazards regression models were then used to estimate hazard ratios (HRs) and 95% confidence intervals (CIs) for the association between tooth loss and mortality outcomes. Tooth loss was considered both as a continuous variable (number of teeth lost) and as a categorical variable (by defined categories of tooth loss). Three models were constructed: Model 1, unadjusted; Model 2, adjusted for age, gender, and race/ethnicity; and Model 3, further adjusted for marital status, education level, BMI, smoking status, COT, diabetes, HT, CVD, and HPL. Trend tests were conducted across ordered tooth loss categories.

Potential nonlinear relationships between the number of lost teeth and mortality were examined using restricted cubic spline (RCS) curves with 4 knots. If evidence of nonlinearity emerged, we conducted a threshold effect analysis using piecewise (segmented) regression and compared models with versus without an inflection point by a likelihood ratio test.

Mediation analysis under the counterfactual framework estimated the average causal mediation effect, average direct effect, total effect, and proportion mediated, accounting for NHANES sampling weights. The 95% CIs for mediation effects were obtained via 1000 bootstrap resamples. To probe potential pathways, we fitted a sequence of models: Model 1 included all covariates; Model 2 additionally adjusted for the FI; and Model 3 further adjusted for log-transformed hs-CRP. We computed the change in log HR (Δlog[HR]) between models and the percentage attenuation: (logHR_M1_ − logHR_Mk_)/logHR_M1_ × 100%. Because hs-CRP may lie on a late segment of the pathway and is temporally proximal, it was not treated as a formal mediator; formal mediation under the counterfactual framework was restricted to the FI.

Additionally, to verify the robustness of the findings, we performed sensitivity analyses: conducted regression analyses for cause-specific mortality to distinguish participants with CVD or cancer, thereby avoiding bias from systemic diseases; performed regression analyses stratified by tooth loss tertiles and compared the results with groupings based on clinical tooth loss categories; repeated regression analyses after excluding subjects who died within the first 2 years of follow-up to avoid bias from early mortality due to comorbid conditions; and re-ran the analysis without multiple imputation to examine the impact of missing values; compared results by excluding participants with missing data directly and retaining only those with complete data; and assessed effect modification by gender, CKD risk strata, and dental prosthetic rehabilitation using multiplicative interaction terms, with stratified estimates reported alongside *P* for interaction.

All statistical analyses were performed using R version 4.5.1 (R Foundation for Statistical Computing) and EmpowerStats (X&Y Solutions, Inc.) software. Two-sided *P* < .05 was considered statistically significant.

## 3. Results

### 3.1. Baseline characteristics

Table 1 details the initial demographic and clinical profiles of CKD patients stratified by survival outcome. The cohort comprised 12,639 adults with CKD (mean age 58.72 ± 16.85 years; male predominance at 64.0%), projecting to a nationally representative cohort of 43.3 million US adults. Based on the degree of tooth loss, participants were categorized as follows: complete dentition (n = 2245, 25%), tooth loss (n = 5029, 42%), lacking functional (n = 2246, 14%), severe tooth loss (n = 1073, 6.4%), and edentulism (n = 2046, 12%). As presented in Table S2, Supplemental Digital Content 2 (categorized by tooth loss), and Table S3, Supplemental Digital Content 3 (tertile-based), edentulous individuals demonstrated significantly higher proportions of advanced age, female sex, lower poverty-income ratios, elevated BMI and waist circumference, and lower educational attainment (high school or below), along with markedly lower rates of lifelong nonsmoking and secondhand smoke avoidance (*P* < .001). Specifically, in the edentulous group, 45% had a history of smoking, 25% were current smokers, and the group had the highest COT levels (80.97 ± 145.70 ng/mL). Compared with other groups, individuals with edentulism had the highest prevalence of CVD (39%) and HPL (68%), while those with severe tooth loss had the highest prevalence of HT (84%) and diabetes (41%). These findings highlight the associations between tooth loss and various baseline characteristics, particularly aging, sex, educational attainment, economic position, nicotine exposure, and multimorbidity patterns.

**Table 1:** Baseline characteristics of the study population.

Characteristic	N*	Overall‡ n† = 43,346,808	Surviving population‡ n† = 32,834,848	Deceased population‡ n† = 10,511,960	*P* value§
Number of missing teeth	12,639	8.29 ± 9.75	6.30 ± 8.54	14.54 ± 10.63	<.001
Tooth loss status¶ (%)	12,639				<.001
Complete dentition		2245 (25)	2044 (30)	201 (6.8)	
Tooth loss		5029 (42)	3833 (45)	1196 (34)	
Lacking functional		2246 (14)	1370 (12)	876 (20)	
Severe tooth loss		1073 (6.4)	564 (4.8)	509 (11)	
Edentulism		2046 (12)	889 (7.6)	1157 (28)	
Age (yr)	12,639	58.72 ± 16.85	54.69 ± 16.26	71.30 ± 11.65	<.001
Age groups (%)	12,639				<.001
0–30		789 (6.6)	776 (8.6)	13 (0.3)	
31–40		844 (8.5)	807 (11)	37 (1.6)	
41–50		1269 (13)	1168 (16)	101 (4.1)	
51–60		1764 (19)	1531 (22)	233 (9.3)	
60–		7973 (53)	4418 (43)	3555 (85)	
Gender (%)	12,639				<.001
Male		8075 (64)	5586 (65)	2489 (58)	
Female		4564 (36)	3114 (35)	1450 (42)	
Race, ethnicity (%)	12,639				<.001
Mexican American		1562 (5.2)	1169 (6.0)	393 (3.0)	
Other Hispanic		784 (4.1)	648 (4.7)	136 (2.5)	
Non-Hispanic White		6117 (71)	3608 (68)	2509 (80)	
Non-Hispanic Black		3317 (14)	2529 (15)	788 (11)	
Other race		859 (5.7)	746 (6.3)	113 (3.6)	
Marital status (%)	12,639				<.001
Married or in a relationship		7419 (64)	5380 (67)	2039 (54)	
Unmarried or single		5220 (36)	3320 (33)	1900 (46)	
PIR	12,639	2.94 ± 1.62	3.10 ± 1.64	2.46 ± 1.47	<.001
PIR categories (%)	12,639				<.001
0–0.9		2390 (13)	1648 (13)	742 (15)	
1.0–2.9		5840 (40)	3675 (35)	2165 (53)	
3.0–5.0		4409 (47)	3377 (52)	1032 (32)	
BMI (kg/m^2^)	12,639	29.41 ± 6.54	29.55 ± 6.50	28.97 ± 6.64	<.001
BMI categories (%)	12,639				<.001
0–18.4		205 (1.6)	131 (1.5)	74 (1.9)	
18.5–24.9		2998 (23)	1889 (22)	1109 (27)	
25.0–29.9		4492 (36)	3071 (36)	1421 (34)	
30.0–		4944 (40)	3609 (41)	1335 (37)	
Waist (cm)	12,639	102.79 ± 16.11	102.51 ± 16.14	103.65 ± 16.00	.012
Smoking status (%)	12,639				<.001
Never smoker		6106 (50)	4501 (53)	1605 (40)	
Current smoker		4328 (33)	2632 (30)	1696 (42)	
Former smoker		2205 (17)	1567 (16)	638 (18)	
Education level (%)	12,639				<.001
Less than high school		3809 (20)	2228 (16)	1581 (32)	
High school or equivalent		3050 (25)	2080 (24)	970 (27)	
College or above		5780 (55)	4392 (59)	1388 (41)	
UACR (mg/g)	12,639	113.91 ± 545.44	86.79 ± 444.03	198.62 ± 775.61	<.001
SCR (mg/dL)	12,639	1.10 ± 0.48	1.07 ± 0.39	1.20 ± 0.68	<.001
ALB (g/L)	12,639	42.25 ± 3.49	42.56 ± 3.36	41.29 ± 3.72	<.001
eGFR (mL/min)	12,639	61.17 ± 23.04	63.92 ± 23.41	52.61 ± 19.47	<.001
HGB (g/dL)	12,639	14.34 ± 1.60	14.47 ± 1.55	13.95 ± 1.71	<.001
COT (ng/mL)	12,639	55.22 ± 130.68	55.15 ± 131.38	55.46 ± 128.49	<.001
HT (%)	12,639				<.001
No		3246 (30)	2647 (35)	599 (16)	
Yes		9393 (70)	6053 (65)	3340 (84)	
HPL (%)	12,639				.009
No		4437 (34)	3107 (35)	1330 (32)	
Yes		8202 (66)	5593 (65)	2609 (68)	
DM (%)	12,639				<.001
No		8746 (74)	6284 (78)	2462 (64)	
Yes		3893 (26)	2416 (22)	1477 (36)	
CVD (%)	12,639				<.001
No		9739 (80)	7320 (86)	2419 (61)	
Yes		2900 (20)	1380 (14)	1520 (39)	
FI	12,639	0.18 ± 0.11	0.16 ± 0.10	0.24 ± 0.13	<.001

ALB = serum albumin, BMI = body mass index, COT = serum cotinine, CVD = cardiovascular disease, DM = diabetes mellitus, eGFR = estimated glomerular filtration rate, FI = Frailty Index, HGB = hemoglobin, HPL = hyperlipidemia, HT = hypertension, PIR = poverty-income ratio, SCR = serum creatinine, SD = standard deviation, UACR = urinary albumin-to-creatinine ratio.

* N refers to the number of participants not missing (unweighted).

† n refers to the number of participants in different categories (weighted).

‡ Mean ± SD; N (%).

§ Design-based Kruskal–Wallis test for continuous variables; Rao–Scott adjusted χ^2^ test for categorical variables.

¶ Specific criteria for tooth loss status: complete dentition (28 teeth), tooth loss (20–27 teeth), lacking functional (9–19 teeth), severe tooth loss (1–8 teeth), and edentulism (without teeth).

### 3.2. The correlation between tooth loss and mortality

**Figure 2.**
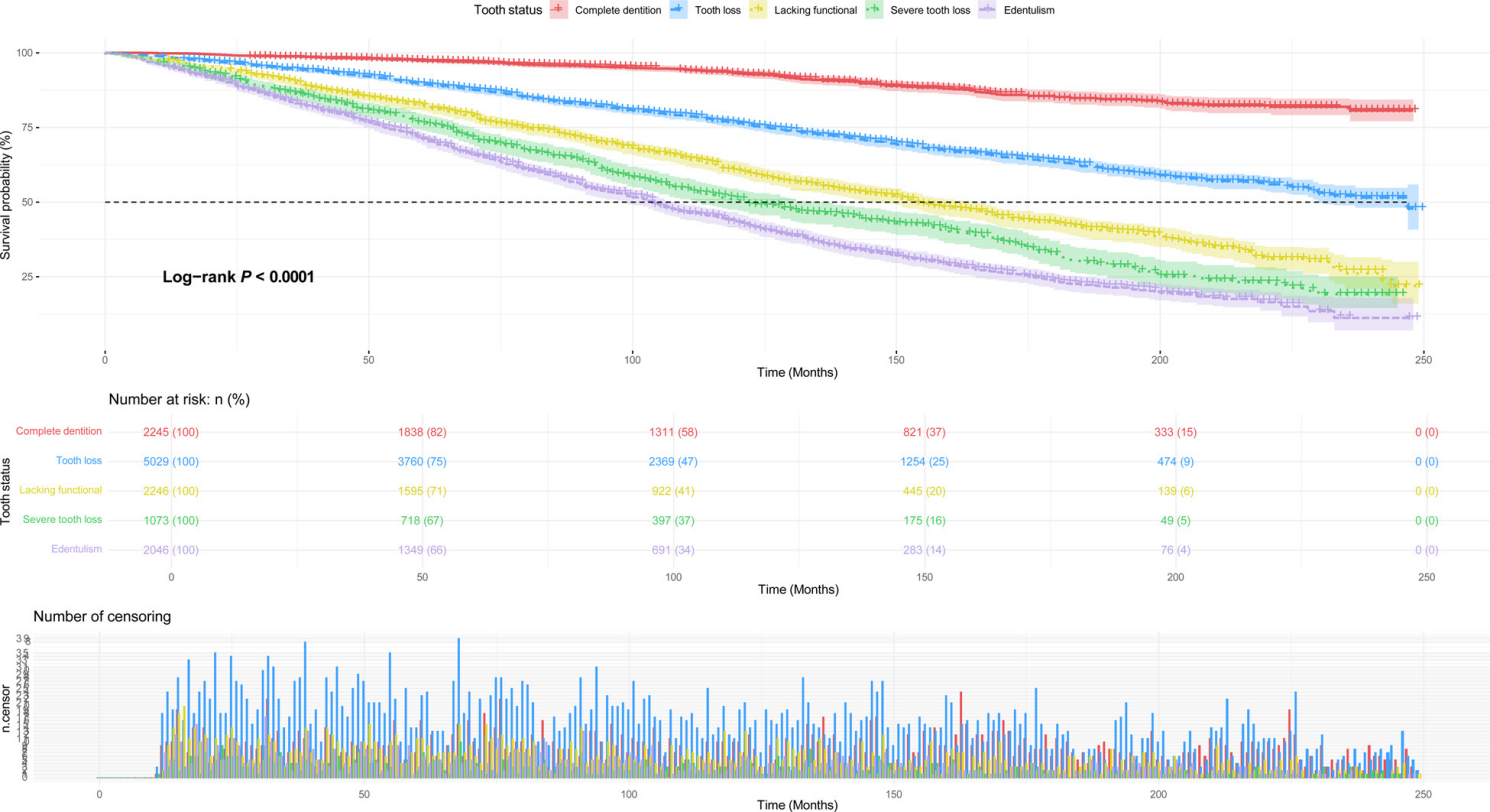
The weighted Kaplan–Meier analysis of tooth loss status with all-cause survival. Specific criteria for tooth status: complete dentition (28 teeth), tooth loss (20–27 teeth), lacking functional (9–19 teeth), severe tooth loss (1–8 teeth), and edentulism (without teeth).

An unadjusted Kaplan–Meier survival analysis (Fig. 2) indicated that there were significant differences in all-cause mortality among CKD patients grouped according to tooth loss status during follow-up (log-rank *P* < .0001). Analyses of cause-specific mortality in CKD patients were consistent with this finding (Figs. S1, S2 and S3, Supplemental Digital Content 4). The lowest all-cause mortality rate was observed in the complete dentate group. As tooth loss increased, mortality rates also gradually increased. Additionally, when assessing all-cause mortality and cause-specific mortality in Cox regression models adjusted for necessary covariates, the results were consistent with the Kaplan–Meier analysis. For all-cause mortality, the unadjusted model (Model 1) HRs and 95% CIs were as follows (in the order of complete dentition, tooth loss, lacking functional, severe tooth loss, and edentulism): 1.00 (reference), 3.21 (2.63–3.93), 6.71 (5.45–8.28), 9.00 (7.16–11.3), and 11.4 (9.48–13.7). In Model 2, after adjusting for age, gender, and race, the HRs and 95% CIs were as follows: 1.00 (reference), 1.72 (1.41–2.11), 2.43 (1.96–3.01), 2.78 (2.19–3.52), and 3.14 (2.58–3.83). In Model 3, after further adjusting for marital status, education level, BMI, smoking status, COT levels, diabetes, HT, CVD, and HPL, the HRs and 95% CIs were as follows: 1.00 (reference), 1.53 (1.24–1.88), 1.93 (1.54–2.42), 1.99 (1.54–2.57), and 2.16 (1.75–2.67), respectively. For the results of multiple Cox regression models for CVD-related mortality, the HRs and 95% CIs for the unadjusted model (Model 1) were as follows: 1.00 (reference), 3.67 (2.49–5.39), 7.85 (5.50–11.2), 12.1 (8.12–18.1), and 13.9 (9.57–20.2). In Model 2, the HRs and 95% CIs were as follows: 1.00 (reference), 1.89 (1.29–2.76), 2.65 (1.85–3.80), 3.47 (2.29–5.26), and 3.55 (2.40–5.25). In Model 3, the HRs and 95% CIs were as follows: 1.00 (reference), 1.62 (1.10–2.40), 2.08 (1.42–3.04), 2.35 (1.53–3.61), and 2.31 (1.54–3.49). For cancer-related mortality, the results of the 3 models also showed a significant positive correlation with increasing tooth loss severity (all *P* < .05 in the 3 models). Additionally, for kidney disease-related mortality, Model 1 initially identified tooth loss count as a mortality predictor (HR: 1.08; CI: 1.06–1.11; *P* < .001). However, multivariable adjustment attenuated this relationship to borderline significance (HR: 1.03; CI: 1.00–1.07; *P* = .070). This lack of significance was consistent in regression analyses stratified by tooth loss status (HR: 3.61; CI: 0.75–17.3; *P* = .284). Tables 2 and 3 present the detailed results of the 3 Cox regression models.

**Table 2:** HR (95% CI) for all-cause mortality by tooth loss count and category from Cox regression.

Model	Tooth loss counts		Tooth loss category					*P* value	*P* for trend
	HR (95% CI)	*P* value	Complete dentition	Tooth loss	Lacking functional	Severe tooth loss	Edentulism		
			HR (95% CI)	HR (95% CI)	HR (95% CI)	HR (95% CI)	HR (95% CI)		
All-cause mortality									
Model 1*	1.07 (1.06–1.07)	<.001	–	3.21 (2.63–3.93)	6.71 (5.45–8.28)	9.00 (7.16–11.3)	11.4 (9.48–13.7)	<.001	<.001
Model 2†	1.03 (1.03–1.03)	<.001	–	1.72 (1.41–2.11)	2.43 (1.96–3.01)	2.78 (2.19–3.52)	3.14 (2.58–3.83)	<.001	<.001
Model 3‡	1.01 (1.01–1.02)	<.001	–	1.53 (1.24–1.88)	1.93 (1.54–2.42)	1.99 (1.54–2.57)	2.16 (1.75–2.67)	<.001	<.001

CI = confidence interval, HR = hazard ratio.

* Model 1: unadjusted.

† Model 2: adjusted for age, gender, and race.

‡ Model 3: adjusted for age, gender, race, marital status, education level, body mass index, smoking status, serum cotinine, diabetes mellitus, hypertension, cardiovascular disease, and hyperlipidemia.

**Table 3:** HR (95% CI) for cause-specific mortality by tooth loss count and category from Cox regression.

Model	Tooth loss counts		Tooth loss category					*P* value	*P* for trend
	HR (95% CI)	*P* value	Complete dentition	Tooth loss	Lacking functional	Severe tooth loss	Edentulism		
			HR (95% CI)	HR (95% CI)	HR (95% CI)	HR (95% CI)	HR (95% CI)		
Cardiovascular disease-related cause									
Model 1*	1.07 (1.07–1.08)	<.001	–	3.67 (2.49–5.39)	7.85 (5.50–11.2)	12.1 (8.12–18.1)	13.9 (9.57–20.2)	<.001	<.001
Model 2†	1.03 (1.03–1.04)	<.001	–	1.89 (1.29–2.76)	2.65 (1.85–3.80)	3.47 (2.29–5.26)	3.55 (2.40–5.25)	<.001	<.001
Model 3‡	1.02 (1.01–1.03)	<.001	–	1.62 (1.10–2.40)	2.08 (1.42–3.04)	2.35 (1.53–3.61)	2.31 (1.54–3.49)	<.001	<.001
Cancer-related cause									
Model 1*	1.06 (1.06–1.07)	<.001	–	2.68 (1.82–3.95)	5.05 (3.31–7.69)	7.19 (4.77–10.8)	9.21 (6.24–13.6)	<.001	<.001
Model 2†	1.03 (1.02–1.04)	<.001	–	1.58 (1.07–2.35)	2.15 (1.36–3.40)	2.67 (1.72–4.12)	3.20 (2.11–4.85)	<.001	<.001
Model 3‡	1.02 (1.01–1.03)	<.001	–	1.39 (0.94–2.04)	1.66 (1.06–2.59)	1.88 (1.23–2.89)	2.16 (1.42–3.29)	<.001	<.001
Kidney disease-related cause									
Model 1*	1.08 (1.06–1.11)	<.001	–	3.91 (1.14–13.4)	10.5 (4.06–27.0)	16.1 (4.38–59.3)	21.7 (6.27–75.1)	<.001	<.001
Model 2†	1.05 (1.02–1.07)	<.001	–	2.12 (0.63–7.16)	3.72 (1.48–9.39)	4.92 (1.41–17.2)	5.98 (1.70–21.0)	.006	<.001
Model 3‡	1.03 (1.00–1.07)	.070	–	1.79 (0.51–6.26)	2.74 (0.99–7.57)	3.11 (0.74–13.0)	3.61 (0.75–17.3)	.284	.085

CI = confidence interval, HR = hazard ratio.

* Model 1: unadjusted.

† Model 2: adjusted for age, gender, and race.

‡ Model 3: adjusted for age, gender, race, marital status, education level, body mass index, smoking status, serum cotinine, diabetes mellitus, hypertension, cardiovascular disease, and hyperlipidemia.

### 3.3. Sensitivity analysis

Stratifying participants by tooth loss tertiles yielded mortality risk patterns for all causes and specific diseases that were consistent with the primary results. In Model 1, using the lowest tertile (T1) as the reference, the HRs and 95% CIs for the second (T2) and highest (T3) tertiles were 2.91 (2.49–3.40) and 7.14 (6.24–8.18), respectively. In Model 2, the HRs (95% CIs) for T2 and T3 were 1.74 (1.51–2.00) and 2.61 (2.27–2.99), respectively. In Model 3, the corresponding values were 1.58 (1.36–1.82) for T2 and 1.97 (1.69–2.29) for T3. Detailed results are presented in Table S4, Supplemental Digital Content 5, of the supplementary materials. In a sensitivity analysis, the exclusion of 24-month mortality cases did not alter the sensitivity analysis inferences. In Model 3, grouping by the number of missing teeth, the HRs (95% CIs) were 1.00 (reference), 1.46 (1.18–1.82), 1.86 (1.48–2.36), 1.88 (1.43–2.47), and 2.04 (1.64–2.54), as shown in Tables S5 and S6, Supplemental Digital Content 7. Supplementary assessments employing datasets retaining original missing values (baseline characteristics in Tables S7 and S8, Supplemental Digital Content 9; regression outputs in Tables S11 and S12, Supplemental Digital Content 11) and analyses confined to participants with complete data (Tables S9 and S10, Supplemental Digital Content 13, for baseline; Tables S13 and S14, Supplemental Digital Content 15, for results) persistently validated the stability of our conclusions.

All subgroup models used the same survey-weighted covariate set as the primary Cox models and, where applicable, were fit within the relevant data subsets. Multiplicative interaction terms were tested, and stratum-specific estimates are reported alongside interaction *P* values in Table S15, Supplemental Digital Content 17. Associations between tooth loss and all-cause mortality were broadly consistent in women and men on both categorical and per-tooth scales. In analyses restricted to cycles with prosthetic information (1999–2004 and 2011–2018), the association between tooth loss and mortality was attenuated among participants with dental prosthetic rehabilitation compared with those without. Interaction results and stratum-specific estimates (none, removable only, fixed/implant only, mixed) are detailed in Table S15, Supplemental Digital Content 17. Among participants reporting prior dialysis, the interaction with dialysis status was not statistically significant, and the overall direction of the association remained aligned with the primary analysis.

In the overall sample, the association between tooth loss and all-cause mortality was observed both among adults without CKD and among those with CKD (*P* for interaction = .041). When risk was further stratified using the KDIGO grid combining eGFR and UACR, the direction of association remained consistent across strata, while the relative effect tended to attenuate in higher-risk categories where baseline hazards were greater (*P* for interaction < .001). Table S16, Supplemental Digital Content 18, provides the specific detailed results.

### 3.4. Nonlinear relationship between tooth loss and all-cause mortality, dose–response relationship

**Figure 3.**
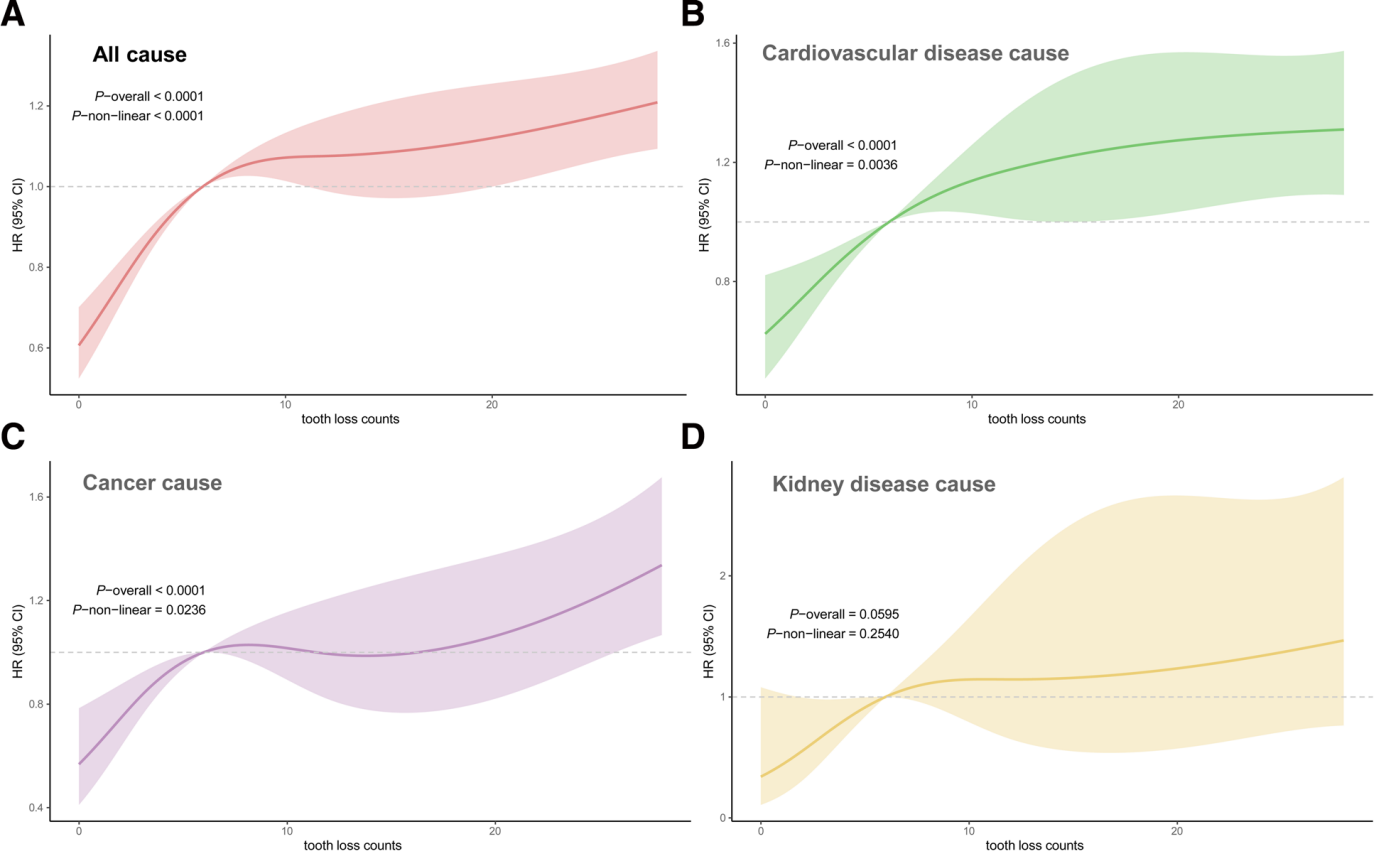
RCS-based associations between tooth loss and mortality in adults with CKD. (A) All-cause mortality; (B) CVD-related mortality; (C) cancer-related mortality; (D) kidney disease-related mortality; HRs were adjusted for age, gender (self-reported male/female), race/ethnicity (Mexican American, Other Hispanic, Non-Hispanic White, Non-Hispanic Black, Other), marital status (married or in a relationship vs unmarried or single), education level (less than high school, high school or equivalent, college or above), BMI, smoking status (current, former, never), serum cotinine, diabetes mellitus, hypertension, cardiovascular disease, and hyperlipidemia. CI = confidence interval, CKD = chronic kidney disease, HR = hazard ratio, RCS = restricted cubic spline.

RCS analysis revealed significant nonlinear relationships between tooth loss counts and all-cause mortality (*P* for nonlinear < .0001), CVD-related mortality (*P* for nonlinear = .0036), and cancer-related mortality (*P* for nonlinear = .0236) in the CKD population. Additionally, no nonlinear association was observed for mortality related to kidney disease (*P* for nonlinear = .2540). In the CKD cohort, a significant upward trend in mortality was observed prior to the RCS inflection points (all-cause mortality inflection point: 6; CVD-related mortality inflection point: 6; cancer-related mortality inflection point: 6). Figure 3 provides more detailed results.

After confirming the presence of a nonlinear association, we employed a piecewise regression model to further explore the dose–response relationship between tooth loss counts and all-cause mortality in the CKD population. After adjusting for potential confounding factors, including age, gender, race, marital status, education level, BMI, smoking status, COT, diabetes mellitus, HT, CVD, and HPL, 3 missing teeth (log-likelihood ratio test, *P* < .001) was determined as the threshold for tooth loss count. Specifically, regardless of whether the tooth loss count was below or above the threshold, it was consistently positively associated with all-cause mortality in the CKD population. When the tooth loss count was below the threshold, the fitted regression model HR was 1.16, with a 95% CI of 1.11 to 1.21 (*P* < .0001); when the tooth loss count exceeded the threshold, the HR from the piecewise regression model was 1.01, with a 95% CI of 1.01 to 1.01 (*P* < .0001). This suggests a potential plateau in the association between the two variables. As the number of missing teeth increased beyond the threshold inflection point, the rate at which the HR increased with the number of missing teeth slowed down. The overall trend of the threshold effect was similar to the curve trend fitted by the RCS model. Table 4 provides the detailed results.

**Table 4:** Threshold effects of tooth loss on all-cause mortality from piecewise Cox regression analysis.

Parameters	Adjusted HR (95% CI)	*P* value
Fitting by the standard linear model*	1.015 (1.012–1.019)	<.0001
Fitting by the two-piecewise linear model*		
Inflection point	3.00	
Tooth loss < 3	1.161 (1.108–1.216)	<.0001
Tooth loss > 3	1.010 (1.006–1.014)	<.0001
Slope 2 − Slope 1	0.870 (0.829–0.913)	<.0001
*P* for likelihood ratio		<.001

CI = confidence interval, HR = hazard ratio.

* Model adjusted for age, gender, race, marital status, education level, body mass index, smoking status, serum cotinine, diabetes mellitus, hypertension, cardiovascular disease, and hyperlipidemia.

### 3.5. The mediating effect of FI and hs-CRP

**Figure 4.**
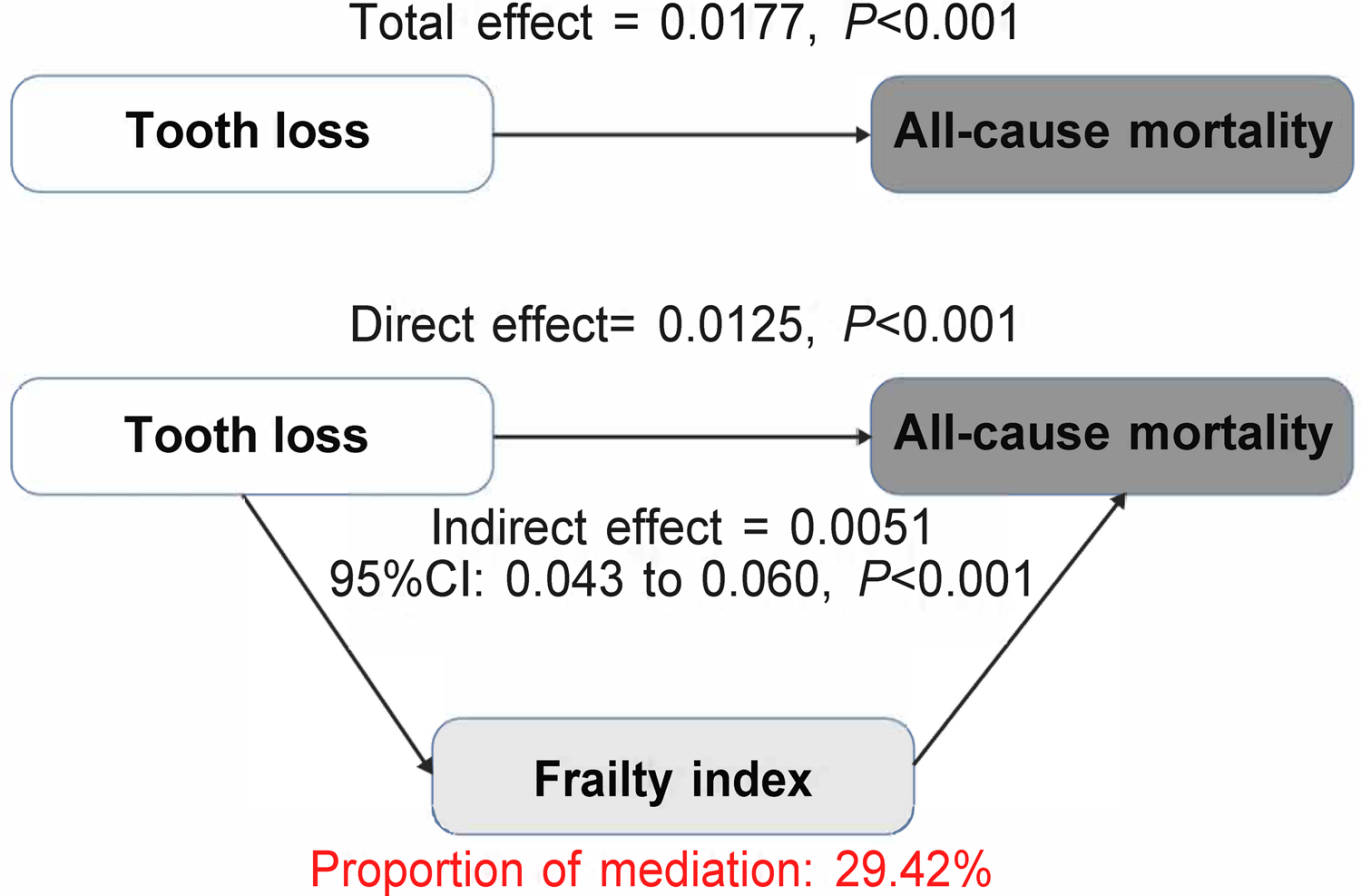
Mediation analysis results. CI = confidence interval.

Mediation analysis revealed that the FI mediated 29.42% of the total effect of missing tooth count on all-cause mortality. The log-transformed indirect effect was 0.0051 (*P* < .001) and the log-transformed direct effect was 0.0125 (*P* < .001; Fig. 4).

In sex-stratified mediation, the proportion mediated by FI was 33.21% in men (total log effect 0.0175, direct 0.0118, indirect 0.0057; 95% CI for the indirect effect 0.005–0.007) and 22.28% in women (total 0.0194, direct 0.0151, indirect 0.0043; 95% CI: 0.003–0.005). Full estimates are shown in Figure S4, Supplemental Digital Content 19.

To probe inflammatory pathways, sequential models adding FI and then ln(hs-CRP) to the fully adjusted Cox model showed stepwise attenuation of the tooth-loss association: per tooth, HR 1.017 → 1.013 → 1.012, with 22.34% attenuation after FI and an additional 4.71% after ln(hs-CRP) (27.05% cumulative); per 10 teeth, HR 1.183 → 1.139 → 1.130, with 22.28% and an additional 4.78% attenuation (cumulative 27.06%). All models remained statistically significant; stepwise HRs are listed in Table S17, Supplemental Digital Content 20, and Δlog(HR)/attenuation metrics are listed in Table S18, Supplemental Digital Content 21. The distribution of hs-CRP and log-transformed hs-CRP values is shown in Figure S5, Supplemental Digital Content 22.

## 4. Discussion

Within this major longitudinal investigation encompassing 12,639 CKD subjects from the serial NHANES surveys from 1999 to 2018 in the United States, we established a significant link between tooth loss and all-cause and cause-specific mortality in adults with CKD. This association remained robust after comprehensive adjustment for potential confounders. Notably, the study revealed that CKD patients with more than 3 missing teeth had a significantly higher risk of all-cause mortality compared to those with fewer missing teeth. More critically, mediation analysis indicated that frailty potentially contributes to this relationship, whereas the contribution of hs-CRP appeared limited. These outcomes were uniformly confirmed through varied sensitivity approaches, buttressing our conclusion that oral health enhancement may improve survival in CKD patients.

In the fully adjusted Cox regression model, using the complete dentate group as the reference, the edentulous group had a 116% higher risk of all-cause mortality, followed by a 99% increase in the severe tooth loss (1–8 teeth) group, a 93% increase in the functional loss (9–19 teeth) group, and a 53% increase in the tooth loss (20–27 teeth) group. Furthermore, tooth loss among CKD patients was also positively associated with cause-specific mortality, including CVD-related and cancer-related deaths. In contrast, the association with kidney disease-related mortality approached significance but did not reach statistical significance. This may be due to objective bias. With the advent and improvement of renal replacement therapies,^[21]^ data from Korea and Sweden have shown that CVD has become the leading cause of death among CKD patients, rather than kidney disease itself,^[22]^ resulting in a shift in the structure of specific causes of death.^[23]^ Since our study did not exclude patients undergoing renal replacement therapy, this may have attenuated the observed association between certain risk factors and kidney disease-related mortality. Additionally, due to discrepancies between ICD-10 code–based identification of CKD and clinical diagnosis pathways,^[24]^ there may be inconsistencies between the definition of kidney disease-related mortality and actual clinical outcomes, potentially underestimating the true correlation. RCS modeling of tooth loss quantity revealed a nonlinear association of missing teeth with all-cause death risk in adults with CKD. Notably, the dose–response analysis revealed a plateau effect in this association. Specifically, with initial tooth loss (<3 teeth), each subsequent tooth loss imposed a 16.1% higher mortality risk. Beyond 3 lost teeth, additional loss contributed only a 1.0% excess risk. This suggests significantly greater mortality risk in the ≥3 missing teeth group versus the <3 teeth group.

In the subgroup analysis, the removable group and the fixed restorations/implants group showed a 1.0% and 1.6% reduction in the risk of death per missing tooth compared with the untreated group. These findings are consistent with multiple cohorts showing lower all-cause (and some cause-specific) mortality among denture users and better survival with implant-supported or well-fitting prostheses, plausibly via improved mastication, nutrition, and reduced systemic inflammation. As for gender, despite similar per-tooth HRs, men showed a larger frailty-mediated proportion than women, with a significant gender interaction. At the same frailty level, men faced a steeper mortality gradient and tended to have greater periodontal burden with lower use of dental and prosthetic care, which amplified functional and inflammatory consequences and limited mitigation by rehabilitation.^[25]^ In CKD, inflammation, hypogonadism, and protein-energy wasting accelerate catabolism and sarcopenia in men, while tooth loss-related declines in chewing efficiency and diet quality further expand the frailty pathway, supporting gender-tailored dental rehabilitation and frailty management.^[26,27]^ When it comes to subgroup analysis of dialysis, limitations such as the small sample size (n = 66; events = 35), the self-reported questionnaire nature, and the inability to distinguish dialysis methods may introduce misclassification and severely limit power. Therefore, estimates should be interpreted cautiously, and we refrain from mechanistic speculation, focusing inference on the more reliable non-dialysis CKD analyses.

Prior research has established tooth loss as a predictor of overall and disease-specific mortality in US national cohorts.^[28]^ Nevertheless, the tooth loss–mortality connection in CKD populations has received scant investigation. Only Ruokonen et al have reported such findings in a study of 144 pre-dialysis CKD patients. In their adjusted Cox model accounting for smoking status, sex, age, diabetic nephropathy, moderate periodontitis, and medication use, tooth deficiency emerged as an independent mortality predictor, which is consistent with our results.^[29]^ While previous studies have similarly examined dose–response effects, the inflection points reported were not entirely consistent with ours. For instance, Holmlund et al found in the general population that individuals with fewer than 25 natural teeth had significantly increased mortality risk compared with those with more than 25.^[30]^ Similarly, a study by Wu et al in cancer survivors showed increased all-cause mortality among those with >5 missing teeth.^[31]^ Shen et al’s analysis revealed that the tooth loss–mortality association exhibited an inverted-L pattern, with mortality risk plateauing once the number of missing teeth reached 10.^[32]^ Therefore, these consistent findings underscore the importance of recognizing the association between tooth loss and mortality risk among CKD patients for both healthcare providers and patients themselves.

The mechanisms by which tooth loss influences mortality risk remain incompletely understood. Our results indicate that frailty accounts for a meaningful share of the tooth loss–mortality association, while systemic inflammation (hs-CRP) explains an additional but smaller share. This pattern is consistent with inflammation being partly embedded within frailty. We have reason to hypothesize that tooth loss may impact mortality in CKD patients by affecting masticatory function, promoting periodontal inflammation, and facilitating microbiota translocation, ultimately leading to frailty. First, multiple missing teeth impair masticatory efficiency, partly through direct effects.^[33]^ Changes in shearing force between the tongue and palate and reduced tongue pressure may contribute to compromised chewing.^[34,35]^ Declining masticatory function increases the likelihood of selecting softer, energy-dense foods (high in saturated fat and sugar)^[36]^ and reduces vitamin intake.^[37]^ The CKD population exhibits baseline vulnerability to protein-energy wasting and micronutrient deficits.^[38]^ Compromised oral function potentially exacerbates cardiovascular event incidence and mortality in this cohort in this way.^[39]^ Second, complex associations between tooth loss, periodontal disease, and CKD have been documented,^[40]^ contributing to mineral-bone disorders,^[41]^ oral inflammation, and dysbiosis,^[42]^ with bidirectional relationships confirmed between them.^[43,44]^ Tooth loss often represents a clinical manifestation of periodontal disease and may serve as a surrogate indicator.^[45–47]^ Periodontitis induces dysbiosis and hyperinflammatory responses in the oral microbiome, leading to systemic effects that elevate premature mortality from various noncommunicable diseases.^[44,48]^ Third, oral health plays a vital role in verbal communication and facial aesthetics.^[49,50]^ Patients with tooth loss may experience “whistling speech” and facial asymmetry or collapse, leading to reduced face-to-face interactions^[50]^ and resulting in social isolation and emotional distress. Numerous studies have confirmed the high prevalence of depression among patients with tooth loss, and the prevalence of depressive symptoms in CKD patients ranges from 20% to 30%.^[51–54]^ Depression in CKD patients often involves hyperactivation of the hypothalamic–pituitary–adrenal axis,^[55]^ increasing cortisol levels and consequently elevating the risks of atherosclerosis, metabolic dysregulation, and infections – all of which contribute to mortality.^[54]^ Depression also impairs self-management and treatment adherence in CKD patients, ultimately leading to adverse outcomes.^[54]^ Finally, tooth loss may contribute to frailty via multiple pathways and negatively impact survival. Impaired chewing due to missing teeth may result in limited dietary variety,^[34,36,37]^ malnutrition, weight loss, and sarcopenia, affecting physical performance.^[9]^ Tooth loss is also closely associated with chronic conditions such as diabetes, HT, CVD,^[38]^ cognitive decline, and depression,^[53]^ which often co-occur and serve as components of the FI.^[9]^ Frailty reflects multisystem degeneration, chronic inflammation, and metabolic imbalance. Through pathways involving malnutrition, increased inflammation, muscle wasting, and social dysfunction, tooth loss may contribute to frailty and increased mortality risk. Therefore, comprehensive interventions targeting these pathways may help reverse or mitigate these adverse effects.

Some key strengths distinguish this investigation. As the inaugural analysis in a nationally representative CKD cohort, it systematically examines tooth loss–mortality associations. To our knowledge, very few studies have addressed this question to date. Second, our exploration of the nonlinear association and inflection point between tooth loss and mortality is novel. Moreover, extensive sensitivity verification ensured outcome resilience. The use of an imputation method designed specifically for the NHANES sampling strategy preserved sampling weights and population representation, enhancing translational relevance to Americans. Although this study elucidated the dose–response mortality pattern for tooth loss and examined the mediation effects of FI, limitations persist. NHANES data constraints precluded definitive causal inference between frailty and tooth loss in mediation models, despite our discussion of their complex interactions. Additionally, time-varying changes in FI could not be assessed. While tooth loss is established as both a core oral health metric and a common proxy for periodontitis/dental caries, the specific etiology of tooth loss remained unidentified in this investigation. Moreover, prosthetic information was available only for some of the cycles, and treatment categories did not capture the timing, quality, or functional performance of restorations. Furthermore, although this study established frailty’s mediation of the tooth loss–mortality association, validating assessments and targeted treatments is imperative to confirm their dual patient-system benefits. Finally, hs-CRP is susceptible to acute-phase variation and limited temporal resolution, so its role was assessed only through sequential modeling.

## Author contributions

**Conceptualization:** Jiaxin Shao, Yiren Bao, Kexin Zhang, Jiazhen Yin, Caifeng Zhu.

**Data curation:** Jiaxin Shao, Yiren Bao.

**Formal analysis:** Jiaxin Shao, Yiren Bao, Kexin Zhang.

**Investigation:** Jiaxin Shao, Yiren Bao, Xinyi Zheng.

**Software:** Jiaxin Shao, Yiren Bao.

**Validation:** Jiaxin Shao, Yiren Bao.

**Writing – original draft:** Jiaxin Shao, Yiren Bao, Kexin Zhang.

**Writing – review & editing:** Xinyi Zheng, Jiazhen Yin, Caifeng Zhu.
















































