# Supplementary material for: Tooth loss elevates all-cause and cause-specific mortality in adults with chronic kidney disease: The mediating role of frailty
Source: Medicine (Baltimore). 2026 Jul 24;105(30):e49843. doi: 10.1097/MD.0000000000049843 (PMC13406305; doi:10.1097/MD.0000000000049843)
Supplement: Supplementary file 1 [file medi-105-e49843-s001.docx]

## **Table S1.** Variables and scoring for frailty index

| Item | | Variable code in NHANES | Scoring (by period) |
| --- | --- | --- | --- |
| **Cognition** | |  |  |
|  | 1. experience confusion/memory problems | PFQ056, PFQ057 | yes=1; no=0 |
| **Dependence** | |  |  |
|  | 2. managing money difficulty | PFQ060a, PFQ061a | no difficulty=0; |
|  |  |  | some difficulty=0.33; |
|  |  |  | much difficulty=0.66; |
|  |  |  | unable to do=1 |
|  | 3. walking for a quarter mile difficulty | PFQ060b, PFQ061b | the same to above |
|  | 4. walking up ten steps difficulty | PFQ060c, PFQ061c | the same to above |
|  | 5. stooping, crouching, kneeling difficulty | PFQ060d, PFQ061d | the same to above |
|  | 6. lifting or carrying difficulty | PFQ060e, PFQ061e | the same to above |
|  | 7. house chore difficulty | PFQ060f, PFQ061f | the same to above |
|  | 8. preparing meals difficulty | PFQ060g, PFQ061g | the same to above |
|  | 9. walking between rooms on same floor | PFQ060h, PFQ061h | the same to above |
|  | 10. standing up from armless chair difficulty | PFQ060i, PFQ061i | the same to above |
|  | 11. getting in and out of bed difficulty | PFQ060j, PFQ061j | the same to above |
|  | 12. using fork, knife, drinking from cup difficulty | PFQ060k, PFQ061k | the same to above |
|  | 13. dressing yourself difficulty | PFQ060l, PFQ061l | the same to above |
|  | 14. standing for long periods difficulty | PFQ060m, PFQ061m | the same to above |
|  | 15. Sitting for long periods difficulty | PFQ060n, PFQ061n | the same to above |
|  | 16. reaching up over head difficulty | PFQ060o, PFQ061o | the same to above |
|  | 17. grasp/holding small objects difficulty | PFQ060p, PFQ061p | the same to above |
|  | 18. going out to movies, events difficulty | PFQ060q, PFQ061q | the same to above |
|  | 19. attending social event difficulty | PFQ060r, PFQ061r | the same to above |
|  | 20. leisure activity at home difficulty | PFQ060s, PFQ061s | the same to above |
|  | 21. push or pull large objects difficulty | PFQ061t | the same to above |
| **Depressive Symptoms** | |  |  |
|  | 22. have little interest in doing things | CIQD008, CIQD009, DPQ010 | **~2004** |
|  |  |  | every day, nearly every day = 1 |
|  |  |  | most days = 0.75 |
|  |  |  | about half the days = 0.50 |
|  |  |  | less than half the days = 0.25 |
|  |  |  |  |
|  |  |  | **2005~** |
|  |  |  | nearly every day = 1 |
|  |  |  | more than half the days = 0.66 |
|  |  |  | several days = 0.33 |
|  | 23. feeling down, depressed, or hopeless | DPQ020, CIQD001, CIQD002 | the same to above |
|  | 24. trouble sleeping or sleeping too much | DPQ030, CIQD025, CIQD026 | **~2004** |
|  |  |  | every night = 1 |
|  |  |  | nearly every night = 0.66 |
|  |  |  | less often = 0.33 |
|  |  |  |  |
|  |  |  | **2005~** |
|  |  |  | nearly every day = 1 |
|  |  |  | more than half the days = 0.66 |
|  |  |  | several days = 0.33 |
|  | 25. feeling tired or having little energy | DPQ040 | nearly every day = 1 |
|  |  |  | more than half the days = 0.66 |
|  |  |  | several days = 0.33 |
|  | 26. poor appetite or overeating | CIQD019, CIQD022, DPQ050 | **~2004** |
|  |  |  | yes = 1 |
|  |  |  | no = 0 |
|  |  |  |  |
|  |  |  | **2005~** |
|  |  |  | the same to above |
|  | 27. feeling bad about yourself | DPQ060, CIQD029 | the same to above |
|  | 28. trouble concentrating on things | DPQ070, CIQD043 | the same to above |
| **Comorbidities** | |  |  |
|  | 29. doctor ever said you had arthritis | MCQ160a | yes = 1; no = 0 |
|  | 30. ever told you had thyroid problem | MCQ160i, MCD160m, MCQ160m | the same to above |
|  | 31. ever told you had chronic bronchitis | MCQ160k, MCQ160p | the same to above |
|  | 32. ever told you had cancer or malignancy | MCQ220 | the same to above |
|  | 33. ever told had congestive heart failure | MCQ160b | the same to above |
|  | 34. ever told you had coronary heart disease | MCQ160c | the same to above |
|  | 35. ever told you had angina/angina pectoris | MCQ160d | the same to above |
|  | 36. ever told you had heart attack | MCQ160e | the same to above |
|  | 37. ever told you had a stroke | MCQ160f | the same to above |
|  | 38. ever told you had high blood pressure | BPQ020 | the same to above |
|  | 39. doctor told you have diabetes | DIQ010 | yes = 1; no =0; borderline=0.5 |
|  | 40. ever told you had weak/failing kidneys | KIQ020, KIQ022 | yes = 1; no =0 |
|  | 41. urine leakage bother you? | KIQ040, KIQ050 | **1999~2000** |
|  |  |  | yes = 1; no = 0 |
|  |  |  |  |
|  |  |  | **2001~** |
|  |  |  | greatly = 1 |
|  |  |  | very much = 0.75 |
|  |  |  | somewhat = 0.5 |
|  |  |  | only a little = 0.25 |
| **Hospital Utilization and Access to Care** | |  |  |
|  | 42. general health condition | HUQ010 | excellent, very good, good = 0 |
|  |  |  | fair, poor = 1 |
|  | 43. health now compared with 1 year ago | HUQ020 | about the same, better = 0 |
|  |  |  | worse = 1 |
|  | 44. overnight hospital patient in last year | HUQ070, HUD070, HUQ071 | yes = 1, no = 0 |
|  | 45. times receive healthcare over past year | HUQ050, HUQ051 | none = 0; 1-4 = 0.5; >=5 =1 |
|  | 46. number of prescription medicines taken | RXD030, RXDUSE, RXD295, RXDCOUNT | no = 0; 1-4 = 0.5; >=5 =1 |
| **Physical Performance and Anthropometry** | | - | - |
|  | 47. body mass index (kg/m^2) | BMXBMI | <18.5, ≥30 = 1 |
|  |  |  | 25–<30 = 0.5 |
|  |  |  | 18.5–25 = 0 |
| **Laboratory Values** | |  |  |
|  | 48. glycohemoglobin (%) | LBXGH | 0%–5.7% = 0, >5.7% = 1 |
|  | 49. red blood cell count (million cells/ul) | LBXRBCSI | Male: 4.7–6.1 = 0, Other = 1 |
|  |  |  | Female: 4.2–5.4 = 0, Other = 1 |
|  | 50. hemoglobin (g/dl) | LBXHGB | Male: 13.5–18 = 0, Other = 1 |
|  |  |  | Female: 12–16 = 0, Other = 1 |
|  | 51. red cell distribution width (%) | LBXRDW | 11.6–14.6 = 0, Other = 1 |
|  | 52. lymphocyte percent (%) | LBXLYPCT | 20–40 = 0, Other = 1 |
|  | 53. segmented neutrophils percent (%) | LBXNEPCT | 40–80 = 0, Other = 1 |
