# Supplementary figures and images for: Tooth loss elevates all-cause and cause-specific mortality in adults with chronic kidney disease: The mediating role of frailty
Source: Medicine (Baltimore). 2026 Jul 24;105(30):e49843. doi: 10.1097/MD.0000000000049843 (PMC13406305; doi:10.1097/MD.0000000000049843)

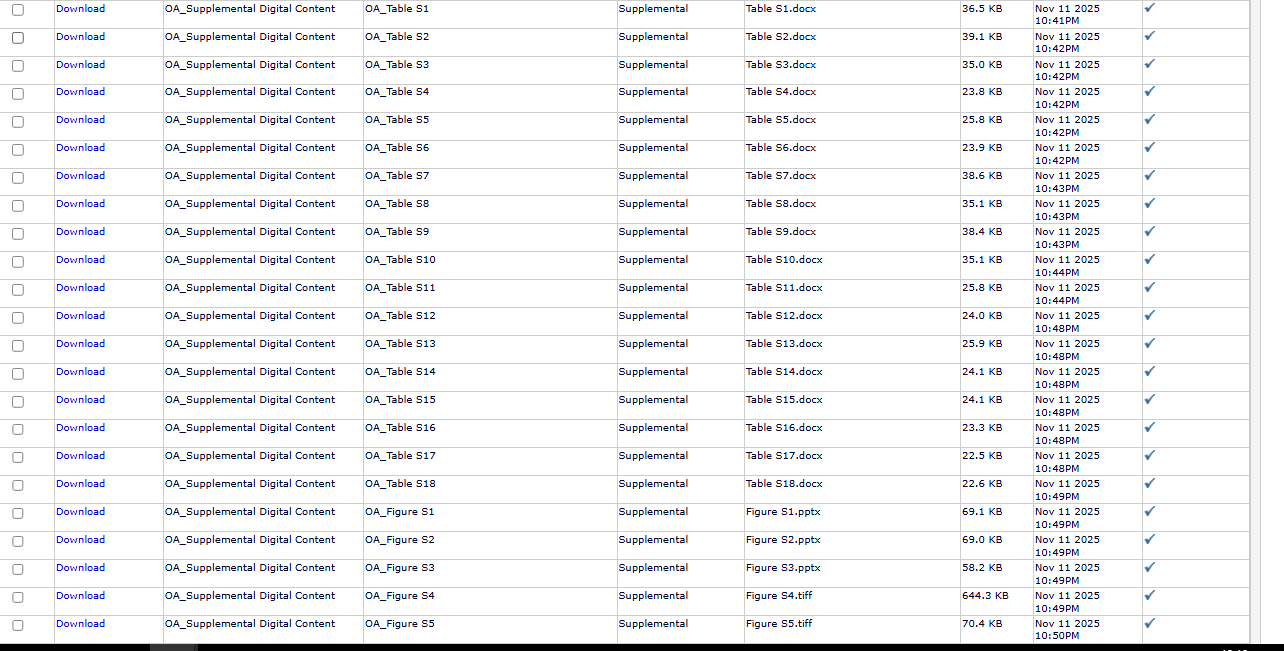

Supplement: Supplementary file 6 [file medi-105-e49843-s006.png]

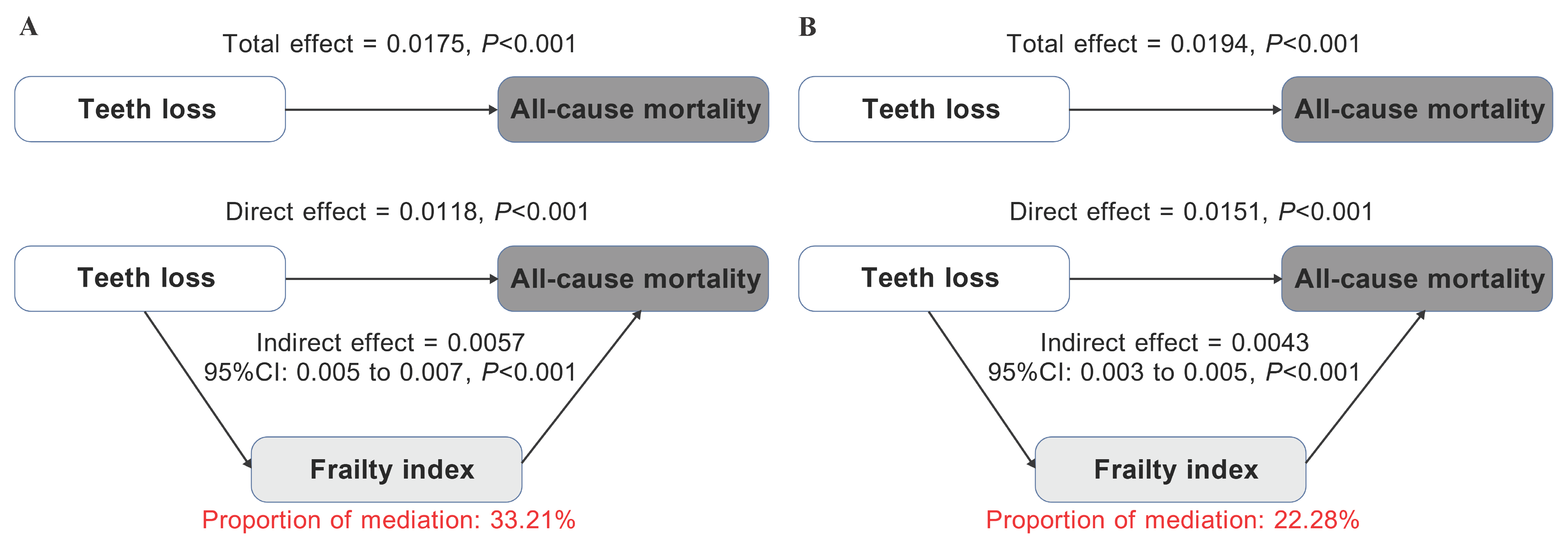

Supplement: Supplementary file 19 [file medi-105-e49843-s019.tiff]

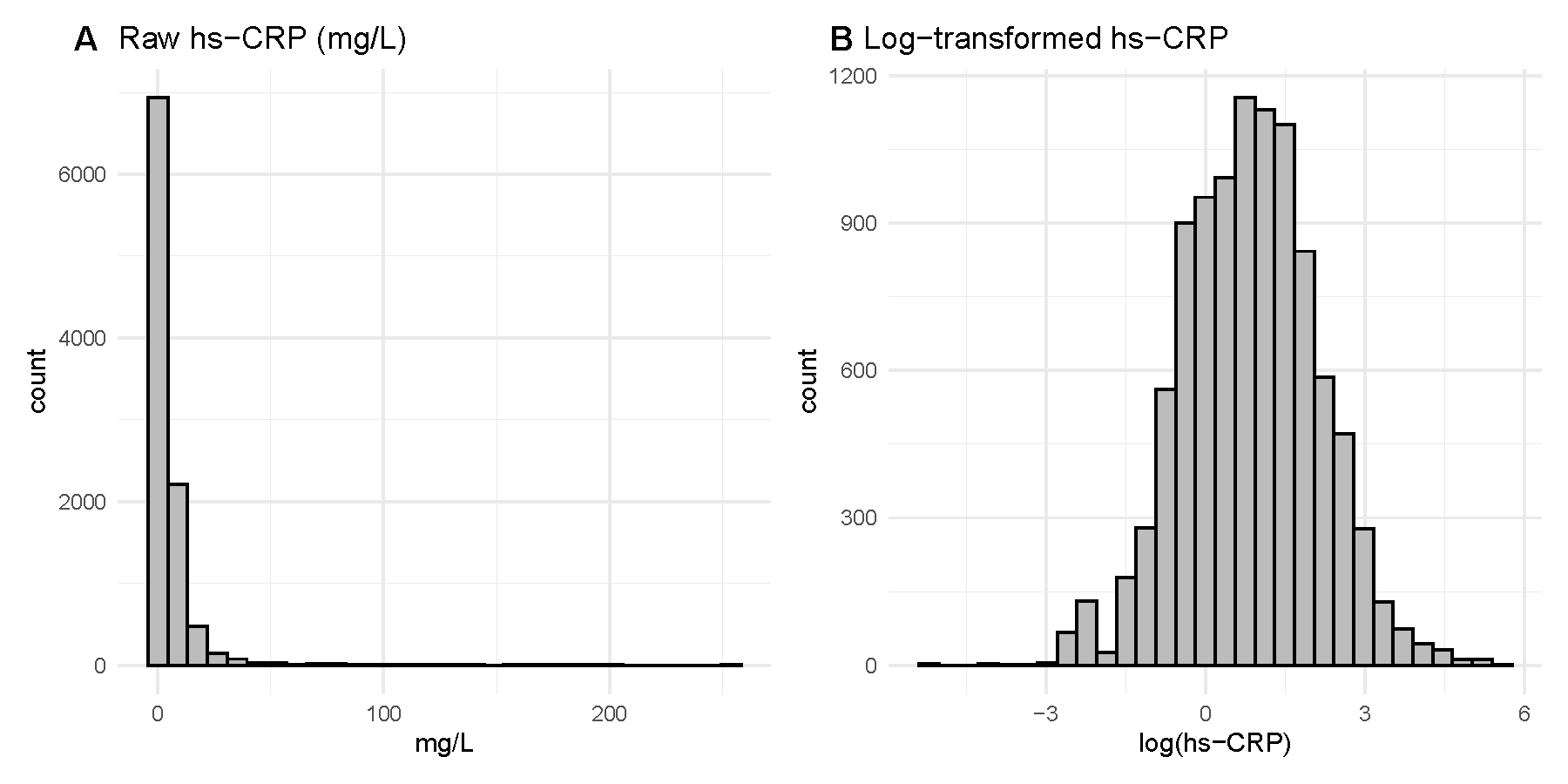

Supplement: Supplementary file 22 [file medi-105-e49843-s022.tiff]
