# Supplementary material for: Tooth loss elevates all-cause and cause-specific mortality in adults with chronic kidney disease: The mediating role of frailty
Source: Medicine (Baltimore). 2026 Jul 24;105(30):e49843. doi: 10.1097/MD.0000000000049843 (PMC13406305; doi:10.1097/MD.0000000000049843)
Supplement: Supplementary file 7 [file medi-105-e49843-s007.docx]

## **Table S5.** HR (95% CIs) for All-cause and cause-specific mortality according to status of tooth loss (duration >2 years)

| **Mortality risk** | **Tooth loss number** |  | **Complete dentition** | **Tooth loss** | **Lacking functional** | **Severe tooth loss** | **Edentulism** |  |  |
| --- | --- | --- | --- | --- | --- | --- | --- | --- | --- |
|  | **HR (95%CI)** | ***P* value** | **HR (95%CI)** | **HR (95%CI)** | **HR (95%CI)** | **HR (95%CI)** | **HR (95%CI)** | ***P* value** | ***P* for trend** |
| **All-cause mortality** | | | | | | | | | |
| Model 1^†^ | 1.07(1.06, 1.07) | <0.001 | — | 3.09(2.51, 3.79) | 6.57(5.30, 8.16) | 8.80(6.89, 11.2) | 11.1(9.16, 13.4) | < .001 | < .001 |
| Model 2^‡^ | 1.03(1.02, 1.03) | <0.001 | — | 1.65(1.34, 2.02) | 2.33(1.88, 2.90) | 2.63(2.05, 3.36) | 2.95(2.41, 3.61) | < .001 | < .001 |
| Model 3^§^ | 1.02(1.01, 1.02) | <0.001 | — | 1.46(1.18, 1.82) | 1.86(1.48, 2.36) | 1.88(1.43, 2.47) | 2.04(1.64, 2.54) | < .001 | < .001 |
| **CVD-related cause** | | | | | | | | | |
| Model 1^†^ | 1.07(1.06, 1.08) | <0.001 | — | 3.60(2.37, 5.48) | 8.08(5.48, 11.9) | 11.9(7.66, 18.5) | 13.2(8.79, 19.7) | < .001 | < .001 |
| Model 2^‡^ | 1.03(1.02, 1.04) | <0.001 | — | 1.83(1.21, 2.78) | 2.65(1.78, 3.92) | 3.27(2.09, 5.11) | 3.23(2.11, 4.93) | < .001 | < .001 |
| Model 3^§^ | 1.02(1.01, 1.02) | <0.001 | — | 1.61(1.05, 2.48) | 2.18(1.43, 3.31) | 2.36(1.46, 3.81) | 2.27(1.44, 3.60) | < .001 | < .001 |
| **Cancer-related cause** | | | | | | | | | |
| Model 1^†^ | 1.07(1.06, 1.07) | <0.001 | — | 2.52(1.67, 3.82) | 4.52(2.92, 6.99) | 7.19(4.68, 11.1) | 8.78(5.81, 13.3) | < .001 | < .001 |
| Model 2^‡^ | 1.03(1.02, 1.04) | <0.001 | — | 1.50(0.98, 2.29) | 1.93(1.19, 3.12) | 2.65(1.67, 4.20) | 3.03(1.94, 4.73) | < .001 | < .001 |
| Model 3^§^ | 1.02(1.01, 1.03) | <0.001 | — | 1.30(0.86, 1.97) | 1.47(0.92, 2.36) | 1.83(1.17, 2.87) | 2.02(1.29, 3.14) | .001 | < .001 |
| **Kidney diseases-related cause** | | | | | | | | | |
| Model 1^†^ | 1.09(1.07, 1.12) | <0.001 | — | 2.95(0.84, 10.4) | 10.5(4.06, 27.0) | 15.0(3.98, 56.7) | 21.0(5.95, 74.0) | < .001 | < .001 |
| Model 2^‡^ | 1.06(1.03, 1.09) | <0.001 | — | 1.59(0.48, 5.29) | 3.18(1.21, 8.36) | 4.44(1.22, 16.1) | 5.64(1.58, 20.1) | .008 | < .001 |
| Model 3^§^ | 1.04(1.00, 1.08) | 0.075 | — | 1.38(0.40, 4.76) | 2.37(0.80, 7.04) | 2.99(0.66, 13.5) | 3.64(0.72, 18.5) | .354 | .113 |

^†^ Model 1: Model unadjusted

^‡^ Model 2: Model adjusted for Age, Gender, Race

^§^ Model 3: Model adjusted for Age, Gender, Race, Marital, Education levels, Body mass index, Smoking status, Serum Cotinine, Diabetes mellitus, Hypertension, Cardiovascular disease, Hyperlipidemia

Abbreviation: HR, hazard ratios; CI, confidence intervals.
