# Supplementary material for: Tooth loss elevates all-cause and cause-specific mortality in adults with chronic kidney disease: The mediating role of frailty
Source: Medicine (Baltimore). 2026 Jul 24;105(30):e49843. doi: 10.1097/MD.0000000000049843 (PMC13406305; doi:10.1097/MD.0000000000049843)
Supplement: Supplementary file 8 [file medi-105-e49843-s008.docx]

## **Table S6.** HR (95% CIs) for All-cause and cause-specific mortality according to tertiles of tooth loss (duration >2 years)

| **Mortality risk** | **T1** | **T2** | **T3** |  |  |
| --- | --- | --- | --- | --- | --- |
|  | **HR (95%CI)** | **HR (95%CI)** | **HR (95%CI)** | ***P* value** | ***P* for trend** |
| **All-cause mortality** | | | | | |
| Model 1^†^ | — | 2.68(2.26, 3.18) | 6.81(5.86, 7.92) | < .001 | < .001 |
| Model 2^‡^ | — | 1.64(1.41, 1.92) | 2.45(2.12, 2.83) | < .001 | < .001 |
| Model 3^§^ | — | 1.51(1.28, 1.77) | 1.86(1.58, 2.19) | < .001 | < .001 |
| **CVD-related cause** | | | | | |
| Model 1^†^ | — | 3.17(2.25, 4.45) | 8.13(6.09, 10.9) | < .001 | < .001 |
| Model 2^‡^ | — | 1.88(1.34, 2.64) | 2.73(2.02, 3.70) | < .001 | < .001 |
| Model 3^§^ | — | 1.72(1.22, 2.42) | 2.14(1.55, 2.94) | < .001 | < .001 |
| **Cancer-related cause** | | | | | |
| Model 1^†^ | — | 2.41(1.67, 3.48) | 5.59(4.01, 7.79) | < .001 | < .001 |
| Model 2^‡^ | — | 1.60(1.11, 2.32) | 2.42(1.68, 3.49) | < .001 | < .001 |
| Model 3^§^ | — | 1.44(1.00, 2.07) | 1.75(1.21, 2.52) | .011 | .002 |
| **Kidney diseases-related cause** | | | | | |
| Model 1^†^ | — | 3.87(1.10, 13.6) | 18.7(6.63, 52.5) | < .001 | < .001 |
| Model 2^‡^ | — | 2.39(0.73, 7.87) | 6.72(2.45, 18.4) | < .001 | < .001 |
| Model 3^§^ | — | 2.14(0.63, 7.29) | 4.87(1.49, 15.9) | .013 | .007 |

^†^ Model 1: Model unadjusted

^‡^ Model 2: Model adjusted for Age, Gender, Race

^§^ Model 3: Model adjusted for Age, Gender, Race, Marital, Education levels, Body mass index, Smoking status, Serum Cotinine, Diabetes mellitus, Hypertension, Cardiovascular disease, Hyperlipidemia

Abbreviation: HR, hazard ratios; CI, confidence intervals.
