# Supplementary material for: Tooth loss elevates all-cause and cause-specific mortality in adults with chronic kidney disease: The mediating role of frailty
Source: Medicine (Baltimore). 2026 Jul 24;105(30):e49843. doi: 10.1097/MD.0000000000049843 (PMC13406305; doi:10.1097/MD.0000000000049843)
Supplement: Supplementary file 9 [file medi-105-e49843-s009.docx]

## **Table S7.** Baseline characteristics of participants of original data with chronic kidney diseases according tooth loss status without multiple interpolation

| **Characteristic** | **N**^*^ | **Overall**^‡^  n^†^ = 43,346,808^2^ | **Complete dentition**  n^†^ = 10,727,724^2^ | **Tooth loss**  n^†^ = 18,378,990^2^ | **Lacking functional**  n^†^ = 6,049,701^2^ | **Severe tooth loss**  n^†^ = 2,776,519^2^ | **Edentulism**  n^†^ = 5,413,874^2^ | ***P*-value**^§^ |
| --- | --- | --- | --- | --- | --- | --- | --- | --- |
| **Age (years)** | 12,639 | 58.72± (16.85) | 46.45± (16.89) | 58.29± (15.57) | 66.08± (12.21) | 68.92± (10.46) | 71.05± (9.95) | < .001 |
| **Age groups (%)** | 12,639 |  |  |  |  |  |  | < .001 |
| 0-30 |  | 789 (6.6%) | 570 (19%) | 213 (4.1%) | 6 (0.5%) | 0 (0%) | 0 (0%) |  |
| 31-40 |  | 844 (8.5%) | 414 (18%) | 389 (8.9%) | 24 (1.3%) | 8 (1.1%) | 9 (0.6%) |  |
| 41-50 |  | 1,269 (13%) | 382 (19%) | 696 (16%) | 138 (8.1%) | 21 (2.7%) | 32 (2.3%) |  |
| 51-60 |  | 1,764 (19%) | 308 (19%) | 883 (22%) | 331 (20%) | 115 (17%) | 127 (10.0%) |  |
| 60- |  | 7,973 (53%) | 571 (25%) | 2,848 (49%) | 1,747 (71%) | 929 (80%) | 1,878 (87%) |  |
| **Gender (%)** | 12,639 |  |  |  |  |  |  | < .001 |
| Male |  | 8,075 (64%) | 1,433 (67%) | 3,299 (65%) | 1,439 (61%) | 695 (62%) | 1,209 (54%) |  |
| Female |  | 4,564 (36%) | 812 (33%) | 1,730 (35%) | 807 (39%) | 378 (38%) | 837 (46%) |  |
| **Race,** **Ethnicity (%)** | 12,639 |  |  |  |  |  |  | < .001 |
| Mexican American |  | 1,562 (5.2%) | 344 (6.3%) | 685 (5.8%) | 273 (4.8%) | 118 (3.8%) | 142 (2.3%) |  |
| Other Hispanic |  | 784 (4.1%) | 119 (3.4%) | 323 (4.6%) | 158 (4.9%) | 66 (3.4%) | 118 (3.4%) |  |
| Non-Hispanic White |  | 6,117 (71%) | 1,106 (74%) | 2,371 (70%) | 953 (64%) | 514 (70%) | 1,173 (75%) |  |
| Non-Hispanic Black |  | 3,317 (14%) | 481 (10%) | 1,268 (13%) | 748 (20%) | 322 (17%) | 498 (13%) |  |
| Other Race |  | 859 (5.7%) | 195 (5.5%) | 382 (5.8%) | 114 (5.8%) | 53 (5.2%) | 115 (5.8%) |  |
| **Marital status (%)** | 12,446 |  |  |  |  |  |  | < .001 |
| Married or in a relationship |  | 7,315 (64%) | 1,349 (68%) | 3,193 (68%) | 1,242 (58%) | 559 (57%) | 972 (52%) |  |
| Unmarried or single |  | 5,131 (36%) | 792 (32%) | 1,778 (32%) | 993 (42%) | 503 (43%) | 1,065 (48%) |  |
| **PIR** | 11,574 | 2.96± (1.62) | 3.47± (1.59) | 3.17± (1.61) | 2.49± (1.47) | 2.28± (1.43) | 2.07± (1.31) | < .001 |
| **PIR categories (%)** | 11,574 |  |  |  |  |  |  | < .001 |
| 0-0.9 |  | 2,179 (13%) | 325 (9.9%) | 721 (11%) | 433 (15%) | 243 (19%) | 457 (20%) |  |
| 1.0-2.9 |  | 5,341 (40%) | 717 (28%) | 1,937 (35%) | 1,081 (51%) | 526 (53%) | 1,080 (59%) |  |
| 3.0-5.0 |  | 4,054 (47%) | 1,037 (62%) | 1,958 (54%) | 543 (33%) | 207 (28%) | 309 (22%) |  |
| **BMI (kg/m^2^)** | 12,404 | 29.38± (6.53) | 28.89± (6.49) | 29.79± (6.75) | 29.32± (6.15) | 29.38± (6.31) | 29.02± (6.30) | < .001 |
| **BMI categories (%)** | 12,404 |  |  |  |  |  |  | < .001 |
| 0-18.4 |  | 198 (1.5%) | 63 (2.5%) | 52 (0.9%) | 30 (1.4%) | 16 (1.0%) | 37 (2.1%) |  |
| 18.5-24.9 |  | 2,955 (23%) | 584 (25%) | 1,096 (21%) | 511 (24%) | 264 (24%) | 500 (24%) |  |
| 25.0-29.9 |  | 4,434 (36%) | 775 (36%) | 1,794 (37%) | 758 (34%) | 372 (33%) | 735 (36%) |  |
| 30.0- |  | 4,817 (40%) | 809 (37%) | 2,022 (41%) | 904 (40%) | 389 (41%) | 693 (38%) |  |
| **Waist (cm)** | 12,071 | 102.55± (15.85) | 99.67± (16.54) | 103.37± (15.74) | 103.32± (14.82) | 104.75± (15.72) | 103.72± (15.23) | < .001 |
| **Smoking status (%)** | 12,396 |  |  |  |  |  |  | < .001 |
| Never smoker |  | 5,972 (50%) | 1,369 (66%) | 2,647 (53%) | 930 (39%) | 377 (33%) | 649 (30%) |  |
| Current smoker |  | 4,311 (34%) | 463 (24%) | 1,581 (32%) | 868 (40%) | 461 (43%) | 938 (45%) |  |
| Former smoker |  | 2,113 (16%) | 231 (10.0%) | 746 (15%) | 443 (21%) | 235 (24%) | 458 (25%) |  |
| **Education levels (%)** | 12,622 |  |  |  |  |  |  | < .001 |
| Less than high school |  | 3,798 (20%) | 356 (8.7%) | 1,164 (15%) | 809 (27%) | 455 (34%) | 1,014 (44%) |  |
| High school or Equivalent |  | 3,047 (25%) | 428 (17%) | 1,185 (24%) | 619 (32%) | 263 (31%) | 552 (31%) |  |
| College or Above |  | 5,777 (55%) | 1,461 (74%) | 2,678 (61%) | 809 (40%) | 353 (35%) | 476 (24%) |  |
| **ACR (mg/g)** | 12,639 | 113.91± (545.44) | 78.22± (386.19) | 108.98± (561.37) | 123.29± (492.89) | 142.07± (560.95) | 176.46± (759.93) | < .001 |
| **SCR (mg/dL)** | 12,639 | 1.10± (0.48) | 1.06± (0.38) | 1.08± (0.48) | 1.14± (0.53) | 1.16± (0.53) | 1.17± (0.59) | < .001 |
| **ALB (g/L)** | 12,639 | 42.25± (3.49) | 43.26± (3.34) | 42.31± (3.43) | 41.66± (3.42) | 41.34± (3.49) | 41.21± (3.56) | < .001 |
| **eGFR (mL/min)** | 12,639 | 61.17± (23.04) | 68.37± (25.60) | 62.24± (22.97) | 55.92± (19.59) | 54.11± (18.48) | 52.81± (17.98) | < .001 |
| **HGB (g/dL)** | 12,615 | 14.34± (1.60) | 14.64± (1.45) | 14.41± (1.59) | 14.09± (1.62) | 14.00± (1.69) | 14.02± (1.74) | < .001 |
| **COT (ng/mL)** | 12,605 | 55.29± (130.76) | 38.53± (114.67) | 49.20± (126.54) | 72.72± (145.38) | 72.16± (138.40) | 81.01± (145.76) | < .001 |
| **Person month (month)** | 12,639 | 102.39± (63.90) | 114.94± (65.73) | 104.57± (64.75) | 94.35± (61.02) | 87.09± (57.50) | 86.98± (57.40) | < .001 |
| **Mortality status (%)** | 12,639 |  |  |  |  |  |  | < .001 |
| 0 |  | 8,700 (76%) | 2,044 (93%) | 3,833 (81%) | 1,370 (65%) | 564 (57%) | 889 (46%) |  |
| 1 |  | 3,939 (24%) | 201 (6.7%) | 1,196 (19%) | 876 (35%) | 509 (43%) | 1,157 (54%) |  |
| **Hypertension (%)** | 12,638 |  |  |  |  |  |  | < .001 |
| No |  | 3,246 (30%) | 1,059 (48%) | 1,312 (29%) | 377 (19%) | 170 (16%) | 328 (17%) |  |
| Yes |  | 9,392 (70%) | 1,186 (52%) | 3,716 (71%) | 1,869 (81%) | 903 (84%) | 1,718 (83%) |  |
| **Hyperlipidemia (%)** | 12,634 |  |  |  |  |  |  | .199 |
| No |  | 4,435 (34%) | 840 (36%) | 1,724 (33%) | 799 (35%) | 387 (34%) | 685 (32%) |  |
| Yes |  | 8,199 (66%) | 1,404 (64%) | 3,305 (67%) | 1,444 (65%) | 686 (66%) | 1,360 (68%) |  |
| **Diabetes (%)** | 12,639 |  |  |  |  |  |  | < .001 |
| No |  | 8,746 (74%) | 1,895 (86%) | 3,579 (75%) | 1,462 (71%) | 611 (59%) | 1,199 (62%) |  |
| Yes |  | 3,893 (26%) | 350 (14%) | 1,450 (25%) | 784 (29%) | 462 (41%) | 847 (38%) |  |
| **CVD (%)** | 12,356 |  |  |  |  |  |  | < .001 |
| No |  | 9,462 (80%) | 1,861 (94%) | 4,014 (83%) | 1,623 (73%) | 707 (66%) | 1,257 (61%) |  |
| Yes |  | 2,894 (20%) | 163 (6.3%) | 953 (17%) | 623 (27%) | 366 (34%) | 789 (39%) |  |
| **FI** | 12,639 | 0.18± (0.11) | 0.13± (0.08) | 0.17± (0.10) | 0.21± (0.12) | 0.23± (0.13) | 0.24± (0.13) | < .001 |

^*^ N refers to number of participants not missing (unweighted)

^†^ n refers to number of participants with different categories (weighted)

^‡^ Mean± (SD); N (%)

^§^ Design-based Kruskal–Wallis test for continuous variables; Rao–Scott adjusted χ² test for categorical variables

Abbreviation: PIR, poverty income ratio; BMI, body mass index; UACR, urinary albumin-to-creatinine ratio; SCR, serum creatinine; ALB, serum albumin; eGFR, estimated glomerular filtration rate; HGB, hemoglobin; COT, serum cotinine; CVD, cardiovascular disease; FI, frailty index.
