# Supplementary material for: Tooth loss elevates all-cause and cause-specific mortality in adults with chronic kidney disease: The mediating role of frailty
Source: Medicine (Baltimore). 2026 Jul 24;105(30):e49843. doi: 10.1097/MD.0000000000049843 (PMC13406305; doi:10.1097/MD.0000000000049843)
Supplement: Supplementary file 10 [file medi-105-e49843-s010.docx]

## **Table S8.** Baseline characteristics of participants of original data with chronic kidney diseases according tooth loss tertiles without multiple interpolation

| **Characteristic** | **N**^*^ | **Overall**^‡^  n^†^ = 43,346,808^2^ | **T1**  n^†^ = 14,650,442^2^ | **T2**  n^†^ = 14,456,271^2^ | **T3**  n^†^ = 14,240,094^2^ | ***P*-value**^§^ |
| --- | --- | --- | --- | --- | --- | --- |
| **Age (years)** | 12,639 | 58.72± (16.85) | 48.72± (17.13) | 59.20± (15.26) | 68.52± (11.28) | < .001 |
| **Age groups (%)** | 12,639 |  |  |  |  | < .001 |
| 0-30 |  | 789 (6.6%) | 643 (16%) | 140 (3.4%) | 6 (0.2%) |  |
| 31-40 |  | 844 (8.5%) | 529 (16%) | 274 (7.9%) | 41 (1.0%) |  |
| 41-50 |  | 1,269 (13%) | 551 (19%) | 527 (15%) | 191 (4.8%) |  |
| 51-60 |  | 1,764 (19%) | 465 (19%) | 726 (22%) | 573 (15%) |  |
| 60- |  | 7,973 (53%) | 1,014 (29%) | 2,405 (51%) | 4,554 (79%) |  |
| **Gender (%)** | 12,639 |  |  |  |  | < .001 |
| Male |  | 8,075 (64%) | 2,080 (68%) | 2,652 (65%) | 3,343 (58%) |  |
| Female |  | 4,564 (36%) | 1,122 (32%) | 1,420 (35%) | 2,022 (42%) |  |
| **Race,** **Ethnicity (%)** | 12,639 |  |  |  |  | < .001 |
| Mexican American |  | 1,562 (5.2%) | 486 (6.3%) | 543 (5.7%) | 533 (3.7%) |  |
| Other Hispanic |  | 784 (4.1%) | 172 (3.8%) | 270 (4.6%) | 342 (4.0%) |  |
| Non-Hispanic White |  | 6,117 (71%) | 1,567 (74%) | 1,910 (70%) | 2,640 (70%) |  |
| Non-Hispanic Black |  | 3,317 (14%) | 691 (11%) | 1,058 (14%) | 1,568 (17%) |  |
| Other Race |  | 859 (5.7%) | 286 (5.8%) | 291 (5.6%) | 282 (5.7%) |  |
| **Marital status (%)** | 12,446 |  |  |  |  | < .001 |
| Married or in a relationship |  | 7,315 (64%) | 1,996 (69%) | 2,546 (67%) | 2,773 (55%) |  |
| Unmarried or single |  | 5,131 (36%) | 1,093 (31%) | 1,477 (33%) | 2,561 (45%) |  |
| **PIR** | 11,574 | 2.96± (1.62) | 3.48± (1.59) | 3.08± (1.60) | 2.29± (1.41) | < .001 |
| **PIR categories (%)** | 11,574 |  |  |  |  | < .001 |
| 0-0.9 |  | 2,179 (13%) | 448 (9.9%) | 598 (12%) | 1,133 (18%) |  |
| 1.0-2.9 |  | 5,341 (40%) | 1,024 (28%) | 1,630 (37%) | 2,687 (54%) |  |
| 3.0-5.0 |  | 4,054 (47%) | 1,479 (62%) | 1,516 (51%) | 1,059 (28%) |  |
| **BMI (kg/m^2^)** | 12,404 | 29.38± (6.53) | 29.08± (6.45) | 29.84± (6.87) | 29.22± (6.24) | .002 |
| **BMI categories (%)** | 12,404 |  |  |  |  | .008 |
| 0-18.4 |  | 198 (1.5%) | 71 (2.0%) | 44 (0.9%) | 83 (1.6%) |  |
| 18.5-24.9 |  | 2,955 (23%) | 794 (23%) | 886 (22%) | 1,275 (24%) |  |
| 25.0-29.9 |  | 4,434 (36%) | 1,125 (37%) | 1,444 (36%) | 1,865 (35%) |  |
| 30.0- |  | 4,817 (40%) | 1,186 (38%) | 1,645 (41%) | 1,986 (40%) |  |
| **Waist (cm)** | 12,071 | 102.55± (15.85) | 100.51± (16.28) | 103.52± (15.86) | 103.75± (15.16) | < .001 |
| **Smoking status (%)** | 12,396 |  |  |  |  | < .001 |
| Never smoker |  | 5,972 (50%) | 1,904 (63%) | 2,112 (52%) | 1,956 (34%) |  |
| Current smoker |  | 4,311 (34%) | 742 (26%) | 1,302 (33%) | 2,267 (42%) |  |
| Former smoker |  | 2,113 (16%) | 357 (11%) | 620 (15%) | 1,136 (23%) |  |
| **Education levels (%)** | 12,622 |  |  |  |  | < .001 |
| Less than high school |  | 3,798 (20%) | 550 (9.8%) | 970 (16%) | 2,278 (35%) |  |
| High school or Equivalent |  | 3,047 (25%) | 628 (18%) | 985 (25%) | 1,434 (32%) |  |
| College or Above |  | 5,777 (55%) | 2,024 (72%) | 2,115 (59%) | 1,638 (33%) |  |
| **ACR (mg/g)** | 12,639 | 113.91± (545.44) | 80.34± (373.62) | 115.18± (608.02) | 147.17± (620.12) | < .001 |
| **SCR (mg/dL)** | 12,639 | 1.10± (0.48) | 1.06± (0.38) | 1.09± (0.50) | 1.16± (0.55) | < .001 |
| **ALB (g/L)** | 12,639 | 42.25± (3.49) | 43.16± (3.37) | 42.15± (3.40) | 41.43± (3.49) | < .001 |
| **eGFR (mL/min)** | 12,639 | 61.17± (23.04) | 67.35± (25.20) | 61.60± (22.68) | 54.39± (18.82) | < .001 |
| **HGB (g/dL)** | 12,615 | 14.34± (1.60) | 14.62± (1.48) | 14.36± (1.59) | 14.04± (1.68) | < .001 |
| **COT (ng/mL)** | 12,605 | 55.29± (130.76) | 39.94± (113.84) | 50.65± (130.28) | 75.76± (144.22) | < .001 |
| **Person month (month)** | 12,639 | 102.39± (63.90) | 113.76± (65.94) | 102.96± (64.20) | 90.13± (59.08) | < .001 |
| **Mortality status (%)** | 12,639 |  |  |  |  | < .001 |
| 0 |  | 8,700 (76%) | 2,846 (92%) | 3,031 (79%) | 2,823 (56%) |  |
| 1 |  | 3,939 (24%) | 356 (8.2%) | 1,041 (21%) | 2,542 (44%) |  |
| **Hypertension (%)** | 12,638 |  |  |  |  | < .001 |
| No |  | 3,246 (30%) | 1,360 (44%) | 1,011 (28%) | 875 (18%) |  |
| Yes |  | 9,392 (70%) | 1,842 (56%) | 3,060 (72%) | 4,490 (82%) |  |
| **Hyperlipidemia (%)** | 12,634 |  |  |  |  | .418 |
| No |  | 4,435 (34%) | 1,164 (35%) | 1,400 (33%) | 1,871 (33%) |  |
| Yes |  | 8,199 (66%) | 2,037 (65%) | 2,672 (67%) | 3,490 (67%) |  |
| **Diabetes (%)** | 12,639 |  |  |  |  | < .001 |
| No |  | 8,746 (74%) | 2,628 (85%) | 2,846 (73%) | 3,272 (65%) |  |
| Yes |  | 3,893 (26%) | 574 (15%) | 1,226 (27%) | 2,093 (35%) |  |
| **CVD (%)** | 12,356 |  |  |  |  | < .001 |
| No |  | 9,462 (80%) | 2,662 (92%) | 3,213 (82%) | 3,587 (67%) |  |
| Yes |  | 2,894 (20%) | 301 (8.0%) | 815 (18%) | 1,778 (33%) |  |
| **FI** | 12,639 | 0.18± (0.11) | 0.13± (0.08) | 0.17± (0.11) | 0.22± (0.12) | < .001 |

^*^ N refers to number of participants not missing (unweighted)

^†^ n refers to number of participants with different categories (weighted)

^‡^ Mean± (SD); N (%)

^§^ Design-based Kruskal–Wallis test for continuous variables; Rao–Scott adjusted χ² test for categorical variables

Abbreviation: PIR, poverty income ratio; BMI, body mass index; UACR, urinary albumin-to-creatinine ratio; SCR, serum creatinine; ALB, serum albumin; eGFR, estimated glomerular filtration rate; HGB, hemoglobin; COT, serum cotinine; CVD, cardiovascular disease; FI, frailty index.
