# Supplementary material for: Tooth loss elevates all-cause and cause-specific mortality in adults with chronic kidney disease: The mediating role of frailty
Source: Medicine (Baltimore). 2026 Jul 24;105(30):e49843. doi: 10.1097/MD.0000000000049843 (PMC13406305; doi:10.1097/MD.0000000000049843)
Supplement: Supplementary file 11 [file medi-105-e49843-s011.docx]

## **Table S11.** HR (95% CIs) for All-cause and cause-specific mortality according to status of tooth loss without multiple interpolation

| **Mortality risk** | **Tooth loss number** |  | **Complete dentition** | **Tooth loss** | **Lacking functional** | **Severe tooth loss** | **Edentulism** |  |  |
| --- | --- | --- | --- | --- | --- | --- | --- | --- | --- |
|  | **HR (95%CI)** | ***P* value** | **HR (95%CI)** | **HR (95%CI)** | **HR (95%CI)** | **HR (95%CI)** | **HR (95%CI)** | ***P* value** | ***P* for trend** |
| **All-cause mortality** | | | | | | | | | |
| Model 1^†^ | 1.07(1.06, 1.07) | <0.001 | — | 3.21(2.63, 3.93) | 6.71(5.45, 8.28) | 9.00(7.16, 11.3) | 11.4(9.48, 13.7) | < .001 | < .001 |
| Model 2^‡^ | 1.03(1.03, 1.03) | <0.001 | — | 1.72(1.41, 2.11) | 2.43(1.96, 3.01) | 2.78(2.19, 3.52) | 3.14(2.58, 3.83) | < .001 | < .001 |
| Model 3^§^ | 1.02(1.01, 1.02) | <0.001 | — | 1.49(1.20, 1.84) | 1.87(1.49, 2.34) | 1.90(1.47, 2.47) | 2.07(1.68, 2.56) | < .001 | < .001 |
| **CVD-related cause** | | | | | | | | | |
| Model 1^†^ | 1.07(1.07, 1.08) | <0.001 | — | 3.67(2.49, 5.39) | 7.85(5.50, 11.2) | 12.1(8.12, 18.1) | 13.9(9.57, 20.2) | < .001 | < .001 |
| Model 2^‡^ | 1.03(1.03, 1.04) | <0.001 | — | 1.89(1.29, 2.76) | 2.65(1.85, 3.80) | 3.47(2.29, 5.26) | 3.55(2.40, 5.25) | < .001 | < .001 |
| Model 3^§^ | 1.02(1.01, 1.03) | <0.001 | — | 1.56(1.05, 2.31) | 1.98(1.35, 2.91) | 2.24(1.45, 3.47) | 2.22(1.47, 3.35) | < .001 | < .001 |
| **Cancer-related cause** | | | | | | | | | |
| Model 1^†^ | 1.06(1.06, 1.07) | <0.001 | — | 2.68(1.82, 3.95) | 5.05(3.31, 7.69) | 7.19(4.77, 10.8) | 9.21(6.24, 13.6) | < .001 | < .001 |
| Model 2^‡^ | 1.03(1.02, 1.04) | <0.001 | — | 1.58(1.07, 2.35) | 2.15(1.36, 3.40) | 2.67(1.72, 4.12) | 3.20(2.11, 4.85) | < .001 | < .001 |
| Model 3^§^ | 1.02(1.01, 1.03) | <0.001 | — | 1.42(0.95, 2.12) | 1.65(1.05, 2.60) | 1.96(1.25, 3.06) | 2.27(1.49, 3.45) | < .001 | < .001 |
| **Kidney diseases-related cause** | | | | | | | | | |
| Model 1^†^ | 1.08(1.06, 1.11) | <0.001 | — | 3.91(1.14, 13.4) | 10.5(4.06, 27.0) | 16.1(4.38, 59.3) | 21.7(6.27, 75.1) | < .001 | < .001 |
| Model 2^‡^ | 1.05(1.02, 1.07) | <0.001 | — | 2.12(0.63, 7.16) | 3.72(1.48, 9.39) | 4.92(1.41, 17.2) | 5.98(1.70, 21.0) | .006 | < .001 |
| Model 3^§^ | 1.03(1.00, 1.07) | 0.083 | — | 1.42(0.42, 4.84) | 2.38(0.85, 6.69) | 2.62(0.62, 11.1) | 3.08(0.65, 14.6) | .372 | .108 |

^†^ Model 1: Model unadjusted

^‡^ Model 2: Model adjusted for Age, Gender, Race

^§^ Model 3: Model adjusted for Age, Gender, Race, Marital, Education levels, Body mass index, Smoking status, Serum Cotinine, Diabetes mellitus, Hypertension, Cardiovascular disease, Hyperlipidemia

Abbreviation: HR, hazard ratios; CI, confidence intervals.
