# Supplementary material for: Tooth loss elevates all-cause and cause-specific mortality in adults with chronic kidney disease: The mediating role of frailty
Source: Medicine (Baltimore). 2026 Jul 24;105(30):e49843. doi: 10.1097/MD.0000000000049843 (PMC13406305; doi:10.1097/MD.0000000000049843)
Supplement: Supplementary file 12 [file medi-105-e49843-s012.docx]

## **Table S12.** HR (95% CIs) for All-cause and cause-specific mortality according to tertiles of tooth loss without multiple interpolation

| **Mortality risk** | **T1** | **T2** | **T3** |  |  |
| --- | --- | --- | --- | --- | --- |
|  | **HR (95%CI)** | **HR (95%CI)** | **HR (95%CI)** | ***P* value** | ***P* for trend** |
| **All-cause mortality** | | | | | |
| Model 1^†^ | — | 2.91(2.49, 3.40) | 7.14(6.24, 8.18) | < .001 | < .001 |
| Model 2^‡^ | — | 1.74(1.51, 2.00) | 2.61(2.27, 2.99) | < .001 | < .001 |
| Model 3^§^ | — | 1.54(1.33, 1.78) | 1.91(1.64, 2.22) | < .001 | < .001 |
| **CVD-related cause** | | | | | |
| Model 1^†^ | — | 3.33(2.43, 4.58) | 8.67(6.57, 11.4) | < .001 | < .001 |
| Model 2^‡^ | — | 1.94(1.41, 2.66) | 2.98(2.23, 3.99) | < .001 | < .001 |
| Model 3^§^ | — | 1.73(1.24, 2.40) | 2.18(1.59, 2.98) | < .001 | < .001 |
| **Cancer-related cause** | | | | | |
| Model 1^†^ | — | 2.69(1.90, 3.79) | 6.18(4.48, 8.52) | < .001 | < .001 |
| Model 2^‡^ | — | 1.74(1.24, 2.44) | 2.68(1.89, 3.80) | < .001 | < .001 |
| Model 3^§^ | — | 1.55(1.09, 2.20) | 1.98(1.38, 2.82) | < .001 | < .001 |
| **Kidney diseases-related cause** | | | | | |
| Model 1^†^ | — | 5.36(1.80, 15.9) | 17.1(6.81, 42.9) | < .001 | < .001 |
| Model 2^‡^ | — | 3.22(1.11, 9.32) | 6.16(2.49, 15.2) | < .001 | < .001 |
| Model 3^§^ | — | 2.32(0.77, 6.95) | 3.79(1.27, 11.3) | .047 | .016 |

^†^ Model 1: Model unadjusted

^‡^ Model 2: Model adjusted for Age, Gender, Race

^§^ Model 3: Model adjusted for Age, Gender, Race, Marital, Education levels, Body mass index, Smoking status, Serum Cotinine, Diabetes mellitus, Hypertension, Cardiovascular disease, Hyperlipidemia

Abbreviation: HR, hazard ratios; CI, confidence intervals.
