# Supplementary material for: Tooth loss elevates all-cause and cause-specific mortality in adults with chronic kidney disease: The mediating role of frailty
Source: Medicine (Baltimore). 2026 Jul 24;105(30):e49843. doi: 10.1097/MD.0000000000049843 (PMC13406305; doi:10.1097/MD.0000000000049843)
Supplement: Supplementary file 13 [file medi-105-e49843-s013.docx]

## **Table S9.** Baseline characteristics of participants of complete data after excluding missing values with chronic kidney diseases according tooth loss status

| **Characteristic** | **N**^*^ | **Overall**^‡^  n^†^ = 37,635,866^2^ | **Complete dentition**  n^†^ = 9,433,612^2^ | **Tooth loss**  n^†^ = 16,193,491^2^ | **Lacking functional**  n^†^ = 5,227,950^2^ | **Severe tooth loss**  n^†^ = 2,348,737^2^ | **Edentulism**  n^†^ = 4,432,076^2^ | ***P*-value**^§^ |
| --- | --- | --- | --- | --- | --- | --- | --- | --- |
| **Age (years)** | 10,620 | 58.68± (16.24) | 47.51± (16.11) | 58.23± (15.19) | 65.77± (12.00) | 68.47± (10.56) | 70.53± (9.96) | < .001 |
| **Age groups (%)** | 10,620 |  |  |  |  |  |  | < .001 |
| 0-30 |  | 449 (5.6%) | 310 (16%) | 135 (3.7%) | 4 (0.4%) | 0 (0%) | 0 (0%) |  |
| 31-40 |  | 757 (8.9%) | 379 (19%) | 344 (8.9%) | 19 (1.2%) | 7 (1.2%) | 8 (0.7%) |  |
| 41-50 |  | 1,147 (14%) | 347 (20%) | 623 (16%) | 126 (8.1%) | 19 (3.0%) | 32 (2.8%) |  |
| 51-60 |  | 1,560 (20%) | 281 (20%) | 784 (22%) | 287 (21%) | 102 (17%) | 106 (9.6%) |  |
| 60- |  | 6,707 (52%) | 508 (25%) | 2,445 (49%) | 1,486 (70%) | 764 (79%) | 1,504 (87%) |  |
| **Gender (%)** | 10,620 |  |  |  |  |  |  | < .001 |
| Male |  | 6,945 (65%) | 1,232 (69%) | 2,882 (66%) | 1,252 (63%) | 580 (62%) | 999 (56%) |  |
| Female |  | 3,675 (35%) | 593 (31%) | 1,449 (34%) | 670 (37%) | 312 (38%) | 651 (44%) |  |
| **Race,** **Ethnicity (%)** | 10,620 |  |  |  |  |  |  | < .001 |
| Mexican American |  | 1,259 (5.0%) | 243 (5.6%) | 572 (5.6%) | 235 (4.9%) | 95 (3.6%) | 114 (2.2%) |  |
| Other Hispanic |  | 622 (3.9%) | 90 (3.2%) | 259 (4.3%) | 134 (4.6%) | 54 (3.4%) | 85 (3.0%) |  |
| Non-Hispanic White |  | 5,279 (72%) | 956 (76%) | 2,082 (71%) | 836 (65%) | 431 (71%) | 974 (76%) |  |
| Non-Hispanic Black |  | 2,761 (14%) | 376 (10%) | 1,100 (13%) | 630 (20%) | 268 (17%) | 387 (13%) |  |
| Other Race |  | 699 (5.5%) | 160 (5.3%) | 318 (5.4%) | 87 (5.6%) | 44 (5.2%) | 90 (6.1%) |  |
| **Marital status (%)** | 10,620 |  |  |  |  |  |  | < .001 |
| Married or in a relationship |  | 6,417 (65%) | 1,215 (69%) | 2,831 (70%) | 1,081 (59%) | 477 (58%) | 813 (54%) |  |
| Unmarried or single |  | 4,203 (35%) | 610 (31%) | 1,500 (30%) | 841 (41%) | 415 (42%) | 837 (46%) |  |
| **PIR** | 10,620 | 3.01± (1.61) | 3.54± (1.56) | 3.20± (1.59) | 2.52± (1.47) | 2.27± (1.41) | 2.11± (1.32) | < .001 |
| **PIR categories (%)** | 10,620 |  |  |  |  |  |  | < .001 |
| 0-0.9 |  | 1,881 (12%) | 227 (8.9%) | 642 (11%) | 395 (15%) | 221 (19%) | 396 (19%) |  |
| 1.0-2.9 |  | 4,885 (39%) | 617 (27%) | 1,814 (35%) | 1,001 (51%) | 483 (54%) | 970 (58%) |  |
| 3.0-5.0 |  | 3,854 (49%) | 981 (64%) | 1,875 (55%) | 526 (34%) | 188 (27%) | 284 (23%) |  |
| **BMI (kg/m^2^)** | 10,620 | 29.39± (6.34) | 29.07± (6.32) | 29.73± (6.47) | 29.30± (6.04) | 29.40± (6.31) | 28.92± (6.20) | .005 |
| **BMI categories (%)** | 10,620 |  |  |  |  |  |  | .016 |
| 0-18.4 |  | 137 (1.3%) | 28 (1.8%) | 39 (0.8%) | 24 (1.3%) | 14 (1.0%) | 32 (2.1%) |  |
| 18.5-24.9 |  | 2,467 (23%) | 430 (23%) | 937 (21%) | 445 (24%) | 223 (24%) | 432 (24%) |  |
| 25.0-29.9 |  | 3,861 (36%) | 666 (37%) | 1,595 (37%) | 676 (35%) | 319 (33%) | 605 (35%) |  |
| 30.0- |  | 4,155 (40%) | 701 (38%) | 1,760 (41%) | 777 (39%) | 336 (42%) | 581 (38%) |  |
| **Waist (cm)** | 10,620 | 102.88± (15.69) | 100.53± (16.27) | 103.54± (15.58) | 103.38± (14.67) | 104.76± (15.88) | 103.86± (15.38) | < .001 |
| **Smoking status (%)** | 10,620 |  |  |  |  |  |  | < .001 |
| Never smoker |  | 5,066 (50%) | 1,209 (66%) | 2,283 (53%) | 768 (37%) | 304 (33%) | 502 (29%) |  |
| Current smoker |  | 3,733 (34%) | 410 (24%) | 1,398 (33%) | 763 (41%) | 394 (44%) | 768 (46%) |  |
| Former smoker |  | 1,821 (16%) | 206 (10%) | 650 (15%) | 391 (22%) | 194 (24%) | 380 (26%) |  |
| **Education levels (%)** | 10,620 |  |  |  |  |  |  | < .001 |
| Less than high school |  | 3,050 (19%) | 234 (7.6%) | 962 (14%) | 680 (26%) | 379 (34%) | 795 (43%) |  |
| High school or Equivalent |  | 2,562 (25%) | 302 (16%) | 1,031 (24%) | 547 (33%) | 221 (32%) | 461 (32%) |  |
| College or Above |  | 5,008 (56%) | 1,289 (77%) | 2,338 (61%) | 695 (41%) | 292 (34%) | 394 (25%) |  |
| **ACR (mg/g)** | 10,620 | 106.39± (523.64) | 76.69± (380.38) | 101.95± (549.34) | 121.74± (494.03) | 124.19± (501.40) | 158.34± (701.78) | < .001 |
| **SCR (mg/dL)** | 10,620 | 1.10± (0.46) | 1.08± (0.38) | 1.08± (0.43) | 1.14± (0.48) | 1.15± (0.50) | 1.18± (0.61) | .002 |
| **ALB (g/L)** | 10,620 | 42.34± (3.38) | 43.25± (3.28) | 42.39± (3.29) | 41.78± (3.34) | 41.39± (3.35) | 41.40± (3.47) | < .001 |
| **eGFR (mL/min)** | 10,620 | 60.74± (22.23) | 66.66± (24.36) | 61.95± (22.33) | 55.78± (19.19) | 54.41± (18.02) | 52.95± (17.78) | < .001 |
| **HGB (g/dL)** | 10,620 | 14.40± (1.58) | 14.67± (1.43) | 14.46± (1.56) | 14.15± (1.61) | 14.02± (1.67) | 14.11± (1.70) | < .001 |
| **COT (ng/mL)** | 10,620 | 56.52± (133.13) | 38.76± (116.91) | 51.01± (130.07) | 73.06± (143.21) | 76.38± (143.95) | 84.45± (149.59) | < .001 |
| **Person month (month)** | 10,620 | 103.79± (63.28) | 113.88± (65.13) | 105.87± (64.01) | 96.76± (60.29) | 90.11± (58.59) | 90.22± (57.71) | < .001 |
| **Mortality status (%)** | 10,620 |  |  |  |  |  |  | < .001 |
| 0 |  | 7,396 (77%) | 1,653 (93%) | 3,336 (82%) | 1,187 (66%) | 478 (59%) | 742 (48%) |  |
| 1 |  | 3,224 (23%) | 172 (6.7%) | 995 (18%) | 735 (34%) | 414 (41%) | 908 (52%) |  |
| **Hypertension (%)** | 10,620 |  |  |  |  |  |  | < .001 |
| No |  | 2,618 (30%) | 766 (45%) | 1,110 (29%) | 330 (19%) | 141 (16%) | 271 (18%) |  |
| Yes |  | 8,002 (70%) | 1,059 (55%) | 3,221 (71%) | 1,592 (81%) | 751 (84%) | 1,379 (82%) |  |
| **Hyperlipidemia (%)** | 10,620 |  |  |  |  |  |  | .609 |
| No |  | 3,697 (34%) | 634 (34%) | 1,504 (34%) | 681 (34%) | 334 (35%) | 544 (32%) |  |
| Yes |  | 6,923 (66%) | 1,191 (66%) | 2,827 (66%) | 1,241 (66%) | 558 (65%) | 1,106 (68%) |  |
| **Diabetes (%)** | 10,620 |  |  |  |  |  |  | < .001 |
| No |  | 7,367 (75%) | 1,520 (86%) | 3,095 (75%) | 1,263 (71%) | 512 (60%) | 977 (62%) |  |
| Yes |  | 3,253 (25%) | 305 (14%) | 1,236 (25%) | 659 (29%) | 380 (40%) | 673 (38%) |  |
| **CVD (%)** | 10,620 |  |  |  |  |  |  | < .001 |
| No |  | 8,219 (81%) | 1,682 (94%) | 3,529 (84%) | 1,397 (73%) | 582 (66%) | 1,029 (63%) |  |
| Yes |  | 2,401 (19%) | 143 (6.2%) | 802 (16%) | 525 (27%) | 310 (34%) | 621 (37%) |  |
| **FI** | 10,620 | 0.17± (0.11) | 0.12± (0.08) | 0.16± (0.10) | 0.20± (0.11) | 0.22± (0.13) | 0.23± (0.12) | < .001 |

^*^ N refers to number of participants not missing (unweighted)

^†^ n refers to number of participants with different categories (weighted)

^‡^ Mean± (SD); N (%)

^§^ Design-based Kruskal–Wallis test for continuous variables; Rao–Scott adjusted χ² test for categorical variables

Abbreviation: PIR, poverty income ratio; BMI, body mass index; UACR, urinary albumin-to-creatinine ratio; SCR, serum creatinine; ALB, serum albumin; eGFR, estimated glomerular filtration rate; HGB, hemoglobin; COT, serum cotinine; CVD, cardiovascular disease; FI, frailty index.
