# Supplementary material for: Tooth loss elevates all-cause and cause-specific mortality in adults with chronic kidney disease: The mediating role of frailty
Source: Medicine (Baltimore). 2026 Jul 24;105(30):e49843. doi: 10.1097/MD.0000000000049843 (PMC13406305; doi:10.1097/MD.0000000000049843)
Supplement: Supplementary file 14 [file medi-105-e49843-s014.docx]

## **Table S10.** Baseline characteristics of participants of complete data after excluding missing values with chronic kidney diseases according tooth loss tertiles

| **Characteristic** | **N**^*^ | **Overall**^‡^  n^†^ = 37,635,866^2^ | **T1**  n^†^ = 12,945,530^2^ | **T2**  n^†^ = 12,681,573^2^ | **T3**  n^†^ = 12,008,763^2^ | ***P*-value**^§^ |
| --- | --- | --- | --- | --- | --- | --- |
| **Age (years)** | 10,620 | 58.68± (16.24) | 49.61± (16.40) | 59.06± (14.90) | 68.05± (11.21) | < .001 |
| **Age groups (%)** | 10,620 |  |  |  |  | < .001 |
| 0-30 |  | 449 (5.6%) | 359 (13%) | 86 (3.1%) | 4 (0.2%) |  |
| 31-40 |  | 757 (8.9%) | 482 (17%) | 241 (7.8%) | 34 (1.0%) |  |
| 41-50 |  | 1,147 (14%) | 495 (20%) | 475 (16%) | 177 (5.1%) |  |
| 51-60 |  | 1,560 (20%) | 415 (20%) | 650 (23%) | 495 (16%) |  |
| 60- |  | 6,707 (52%) | 893 (30%) | 2,060 (50%) | 3,754 (78%) |  |
| **Gender (%)** | 10,620 |  |  |  |  | < .001 |
| Male |  | 6,945 (65%) | 1,795 (69%) | 2,319 (65%) | 2,831 (60%) |  |
| Female |  | 3,675 (35%) | 849 (31%) | 1,193 (35%) | 1,633 (40%) |  |
| **Race,** **Ethnicity (%)** | 10,620 |  |  |  |  | < .001 |
| Mexican American |  | 1,259 (5.0%) | 361 (5.7%) | 454 (5.5%) | 444 (3.6%) |  |
| Other Hispanic |  | 622 (3.9%) | 132 (3.5%) | 217 (4.2%) | 273 (3.8%) |  |
| Non-Hispanic White |  | 5,279 (72%) | 1,367 (75%) | 1,671 (71%) | 2,241 (70%) |  |
| Non-Hispanic Black |  | 2,761 (14%) | 551 (10%) | 925 (14%) | 1,285 (17%) |  |
| Other Race |  | 699 (5.5%) | 233 (5.5%) | 245 (5.2%) | 221 (5.7%) |  |
| **Marital status (%)** | 10,620 |  |  |  |  | < .001 |
| Married or in a relationship |  | 6,417 (65%) | 1,788 (71%) | 2,258 (68%) | 2,371 (57%) |  |
| Unmarried or single |  | 4,203 (35%) | 856 (29%) | 1,254 (32%) | 2,093 (43%) |  |
| **PIR** | 10,620 | 3.01± (1.61) | 3.53± (1.57) | 3.12± (1.59) | 2.32± (1.42) | < .001 |
| **PIR categories (%)** | 10,620 |  |  |  |  | < .001 |
| 0-0.9 |  | 1,881 (12%) | 338 (9.1%) | 531 (11%) | 1,012 (17%) |  |
| 1.0-2.9 |  | 4,885 (39%) | 901 (27%) | 1,530 (37%) | 2,454 (54%) |  |
| 3.0-5.0 |  | 3,854 (49%) | 1,405 (64%) | 1,451 (52%) | 998 (29%) |  |
| **BMI (kg/m^2^)** | 10,620 | 29.39± (6.34) | 29.23± (6.29) | 29.75± (6.55) | 29.18± (6.15) | .031 |
| **BMI categories (%)** | 10,620 |  |  |  |  | .079 |
| 0-18.4 |  | 137 (1.3%) | 33 (1.5%) | 34 (0.9%) | 70 (1.5%) |  |
| 18.5-24.9 |  | 2,467 (23%) | 603 (22%) | 764 (21%) | 1,100 (24%) |  |
| 25.0-29.9 |  | 3,861 (36%) | 979 (37%) | 1,282 (37%) | 1,600 (35%) |  |
| 30.0- |  | 4,155 (40%) | 1,029 (39%) | 1,432 (41%) | 1,694 (39%) |  |
| **Waist (cm)** | 10,620 | 102.88± (15.69) | 101.28± (16.05) | 103.60± (15.67) | 103.83± (15.18) | < .001 |
| **Smoking status (%)** | 10,620 |  |  |  |  | < .001 |
| Never smoker |  | 5,066 (50%) | 1,664 (63%) | 1,828 (52%) | 1,574 (33%) |  |
| Current smoker |  | 3,733 (34%) | 661 (26%) | 1,147 (33%) | 1,925 (43%) |  |
| Former smoker |  | 1,821 (16%) | 319 (11%) | 537 (15%) | 965 (24%) |  |
| **Education levels (%)** | 10,620 |  |  |  |  | < .001 |
| Less than high school |  | 3,050 (19%) | 392 (8.8%) | 804 (15%) | 1,854 (34%) |  |
| High school or Equivalent |  | 2,562 (25%) | 470 (17%) | 863 (26%) | 1,229 (33%) |  |
| College or Above |  | 5,008 (56%) | 1,782 (74%) | 1,845 (59%) | 1,381 (33%) |  |
| **ACR (mg/g)** | 10,620 | 106.39± (523.64) | 76.63± (355.28) | 109.00± (603.23) | 135.72± (580.81) | < .001 |
| **SCR (mg/dL)** | 10,620 | 1.10± (0.46) | 1.07± (0.37) | 1.08± (0.45) | 1.15± (0.53) | < .001 |
| **ALB (g/L)** | 10,620 | 42.34± (3.38) | 43.17± (3.31) | 42.24± (3.25) | 41.57± (3.40) | < .001 |
| **eGFR (mL/min)** | 10,620 | 60.74± (22.23) | 65.91± (24.06) | 61.42± (22.07) | 54.47± (18.49) | < .001 |
| **HGB (g/dL)** | 10,620 | 14.40± (1.58) | 14.65± (1.47) | 14.42± (1.56) | 14.11± (1.66) | < .001 |
| **COT (ng/mL)** | 10,620 | 56.52± (133.13) | 40.16± (116.06) | 52.97± (134.20) | 77.91± (145.80) | < .001 |
| **Person month (month)** | 10,620 | 103.79± (63.28) | 113.06± (65.33) | 104.50± (63.43) | 93.05± (59.10) | < .001 |
| **Mortality status (%)** | 10,620 |  |  |  |  | < .001 |
| 0 |  | 7,396 (77%) | 2,342 (92%) | 2,647 (80%) | 2,407 (58%) |  |
| 1 |  | 3,224 (23%) | 302 (7.8%) | 865 (20%) | 2,057 (42%) |  |
| **Hypertension (%)** | 10,620 |  |  |  |  | < .001 |
| No |  | 2,618 (30%) | 1,019 (42%) | 857 (28%) | 742 (18%) |  |
| Yes |  | 8,002 (70%) | 1,625 (58%) | 2,655 (72%) | 3,722 (82%) |  |
| **Hyperlipidemia (%)** | 10,620 |  |  |  |  | .756 |
| No |  | 3,697 (34%) | 914 (34%) | 1,224 (34%) | 1,559 (33%) |  |
| Yes |  | 6,923 (66%) | 1,730 (66%) | 2,288 (66%) | 2,905 (67%) |  |
| **Diabetes (%)** | 10,620 |  |  |  |  | < .001 |
| No |  | 7,367 (75%) | 2,156 (85%) | 2,459 (74%) | 2,752 (66%) |  |
| Yes |  | 3,253 (25%) | 488 (15%) | 1,053 (26%) | 1,712 (34%) |  |
| **CVD (%)** | 10,620 |  |  |  |  | < .001 |
| No |  | 8,219 (81%) | 2,383 (92%) | 2,828 (82%) | 3,008 (68%) |  |
| Yes |  | 2,401 (19%) | 261 (7.8%) | 684 (18%) | 1,456 (32%) |  |
| **FI** | 10,620 | 0.17± (0.11) | 0.13± (0.08) | 0.17± (0.10) | 0.22± (0.12) | < .001 |

^*^ N refers to number of participants not missing (unweighted)

^†^ n refers to number of participants with different categories (weighted)

^‡^ Mean± (SD); N (%)

^§^ Design-based Kruskal–Wallis test for continuous variables; Rao–Scott adjusted χ² test for categorical variables

Abbreviation: PIR, poverty income ratio; BMI, body mass index; UACR, urinary albumin-to-creatinine ratio; SCR, serum creatinine; ALB, serum albumin; eGFR, estimated glomerular filtration rate; HGB, hemoglobin; COT, serum cotinine; CVD, cardiovascular disease; FI, frailty index.
