# Supplementary material for: Tooth loss elevates all-cause and cause-specific mortality in adults with chronic kidney disease: The mediating role of frailty
Source: Medicine (Baltimore). 2026 Jul 24;105(30):e49843. doi: 10.1097/MD.0000000000049843 (PMC13406305; doi:10.1097/MD.0000000000049843)
Supplement: Supplementary file 15 [file medi-105-e49843-s015.docx]

## **Table S13.** The results of the sensitivity analysis of complete data after excluding missing values according tooth loss status

| **Mortality risk** | **Tooth loss number** |  | **Complete dentition** | **Tooth loss** | **Lacking functional** | **Severe tooth loss** | **Edentulism** |  |  |
| --- | --- | --- | --- | --- | --- | --- | --- | --- | --- |
|  | **HR (95%CI)** | ***P* value** | **HR (95%CI)** | **HR (95%CI)** | **HR (95%CI)** | **HR (95%CI)** | **HR (95%CI)** | ***P* value** | ***P* for trend** |
| **All-cause mortality** | | | | | | | | | |
| Model 1^†^ | 1.07(1.06, 1.07) | < .001 | — | 2.95(2.38, 3.65) | 6.33(5.05, 7.93) | 8.27(6.41, 10.7) | 10.6(8.60, 13.0) | < .001 | < .001 |
| Model 2^‡^ | 1.03(1.03, 1.03) | < .001 | — | 1.60(1.29, 1.98) | 2.30(1.84, 2.88) | 2.57(1.99, 3.34) | 2.99(2.42, 3.71) | < .001 | < .001 |
| Model 3^§^ | 1.02(1.01, 1.02) | < .001 | — | 1.41(1.13, 1.75) | 1.81(1.42, 2.29) | 1.83(1.38, 2.41) | 2.05(1.63, 2.57) | < .001 | < .001 |
| **CVD-related cause** | | | | | | | | | |
| Model 1^†^ | 1.07(1.06, 1.08) | < .001 | — | 3.23(2.18, 4.81) | 6.88(4.71, 10.1) | 11.3(7.35, 17.3) | 12.4(8.40, 18.2) | < .001 | < .001 |
| Model 2^‡^ | 1.03(1.02, 1.04) | < .001 | — | 1.67(1.12, 2.49) | 2.33(1.58, 3.44) | 3.26(2.09, 5.09) | 3.27(2.14, 4.97) | < .001 | < .001 |
| Model 3^§^ | 1.02(1.01, 1.03) | < .001 | — | 1.42(0.95, 2.13) | 1.81(1.21, 2.70) | 2.18(1.40, 3.40) | 2.10(1.37, 3.20) | < .001 | < .001 |
| **Cancer-related cause** | | | | | | | | | |
| Model 1^†^ | 1.06(1.05, 1.07) | < .001 | — | 2.48(1.65, 3.72) | 4.49(2.88, 7.02) | 6.54(4.22, 10.1) | 8.35(5.51, 12.6) | < .001 | < .001 |
| Model 2^‡^ | 1.03(1.02, 1.04) | < .001 | — | 1.47(0.98, 2.22) | 1.91(1.19, 3.06) | 2.44(1.55, 3.86) | 2.92(1.91, 4.48) | < .001 | < .001 |
| Model 3^§^ | 1.02(1.01, 1.03) | < .001 | — | 1.30(0.87, 1.95) | 1.48(0.93, 2.36) | 1.76(1.11, 2.79) | 2.01(1.30, 3.11) | .002 | < .001 |
| **Kidney diseases-related cause** | | | | | | | | | |
| Model 1^†^ | 1.10(1.07, 1.13) | < .001 | — | 4.09(0.88, 19.1) | 14.8(2.96, 73.5) | 19.9(4.07, 97.1) | 34.0(7.39, 157) | < .001 | < .001 |
| Model 2^‡^ | 1.07(1.03, 1.10) | < .001 | — | 2.32(0.51, 10.6) | 5.49(1.00, 30.2) | 6.41(1.26, 32.8) | 10.2(2.05, 51.2) | .006 | < .001 |
| Model 3^§^ | 1.05(1.01, 1.09) | .018 | — | 1.92(0.42, 8.70) | 3.78(0.70, 20.5) | 3.89(0.74, 20.5) | 5.71(0.98, 33.1) | .217 | .026 |

^†^ Model 1: Model unadjusted

^‡^ Model 2: Model adjusted for Age, Gender, Race

^§^ Model 3: Model adjusted for Age, Gender, Race, Marital, Education levels, Body mass index, Smoking status, Serum Cotinine, Diabetes mellitus, Hypertension, Cardiovascular disease, Hyperlipidemia

Abbreviation: HR, hazard ratios; CI, confidence intervals.
