# Supplementary material for: Tooth loss elevates all-cause and cause-specific mortality in adults with chronic kidney disease: The mediating role of frailty
Source: Medicine (Baltimore). 2026 Jul 24;105(30):e49843. doi: 10.1097/MD.0000000000049843 (PMC13406305; doi:10.1097/MD.0000000000049843)
Supplement: Supplementary file 16 [file medi-105-e49843-s016.docx]

## **Table S14.** The results of the sensitivity analysis of complete data after excluding missing values according tooth loss tertiles

| **Mortality risk** | **T1** | **T2** | **T3** |  |  |
| --- | --- | --- | --- | --- | --- |
|  | **HR (95%CI)** | **HR (95%CI)** | **HR (95%CI)** | ***P* value** | ***P* for trend** |
| **All-cause mortality** | | | | | |
| Model 1^†^ | — | 2.81(2.37, 3.34) | 6.93(5.91, 8.14) | < .001 | < .001 |
| Model 2^‡^ | — | 1.71(1.47, 2.00) | 2.59(2.22, 3.02) | < .001 | < .001 |
| Model 3^§^ | — | 1.53(1.31, 1.79) | 1.94(1.64, 2.30) | < .001 | < .001 |
| **CVD-related cause** | | | | | |
| Model 1^†^ | — | 3.11(2.22, 4.35) | 8.13(6.03, 11.0) | < .001 | < .001 |
| Model 2^‡^ | — | 1.84(1.31, 2.56) | 2.87(2.09, 3.95) | < .001 | < .001 |
| Model 3^§^ | — | 1.61(1.15, 2.26) | 2.09(1.52, 2.88) | < .001 | < .001 |
| **Cancer-related cause** | | | | | |
| Model 1^†^ | — | 2.49(1.72, 3.60) | 5.59(3.94, 7.95) | < .001 | < .001 |
| Model 2^‡^ | — | 1.64(1.14, 2.34) | 2.45(1.70, 3.53) | < .001 | < .001 |
| Model 3^§^ | — | 1.47(1.03, 2.10) | 1.82(1.25, 2.64) | .007 | .001 |
| **Kidney diseases-related cause** | | | | | |
| Model 1^†^ | — | 7.27(1.55, 34.2) | 30.6(6.97, 134) | < .001 | < .001 |
| Model 2^‡^ | — | 4.53(0.99, 20.8) | 11.7(2.51, 54.2) | .002 | < .001 |
| Model 3^§^ | — | 3.80(0.84, 17.2) | 7.33(1.54, 34.9) | .030 | .006 |

^†^ Model 1: Model unadjusted

^‡^ Model 2: Model adjusted for Age, Gender, Race

^§^ Model 3: Model adjusted for Age, Gender, Race, Marital, Education levels, Body mass index, Smoking status, Serum Cotinine, Diabetes mellitus, Hypertension, Cardiovascular disease, Hyperlipidemia

Abbreviation: HR, hazard ratios; CI, confidence intervals.
