# Supplementary material for: Tooth loss elevates all-cause and cause-specific mortality in adults with chronic kidney disease: The mediating role of frailty
Source: Medicine (Baltimore). 2026 Jul 24;105(30):e49843. doi: 10.1097/MD.0000000000049843 (PMC13406305; doi:10.1097/MD.0000000000049843)
Supplement: Supplementary file 17 [file medi-105-e49843-s017.docx]

## **Table S15.** Subgroup analysis of variables in the available period

| **Variates** | **n**^†^ | **Event**^‡^ | **HR (95%CI)** | ***P* for interaction** |
| --- | --- | --- | --- | --- |
| **Gender** | | | | .018 |
| Male | 8075 | 2489 | 1.017(1.012, 1.022) |  |
| Female | 4564 | 1450 | 1.017(1.011, 1.023) |  |
| **Dialysis** | | | | .211 |
| No | 11531 | 3420 | 1.017(1.013, 1.021) |  |
| Yes | 66 | 35 | 0.977(0.925, 1.032) |  |
| **Dental Treatment** | | | | .037 |
| No | 6385 | 1609 | 1.016(1.010, 1.023) |  |
| Yes | 3465 | 1266 | 1.015(1.008, 1.022) |  |
| **Dental Treatment (classification of treatment methods)** | | | | .004 |
| None | 4008 | 637 | 1.028(1.014, 1.041) |  |
| Removable only | 1985 | 683 | 1.018(1.006, 1.030) |  |
| Fixed/Implant only | 1228 | 494 | 1.012(1.001, 1.024) |  |
| Mixed | 212 | 77 | 0.977(0.940, 1.016) |  |

^†^ n refers to number of participants with different categories (unweighted)

^‡^ Event refers to number of all cause death events for participants with different categories (unweighted)

Subgroup analysis conducted on gender, dialysis status, dental treatment (no/yes), and dental treatment (classification of treatment methods) within each subgroup variable available period.

Model adjusted for Age, Gender, Race, Marital, Education levels, Body mass index, Smoking status, Serum Cotinine, Diabetes mellitus, Hypertension, Cardiovascular disease, Hyperlipidemia. But the model did not adjust for the stratification variables themselves.

Abbreviation: HR, hazard ratios; CI, confidence intervals.
