# Supplementary material for: Tooth loss elevates all-cause and cause-specific mortality in adults with chronic kidney disease: The mediating role of frailty
Source: Medicine (Baltimore). 2026 Jul 24;105(30):e49843. doi: 10.1097/MD.0000000000049843 (PMC13406305; doi:10.1097/MD.0000000000049843)
Supplement: Supplementary file 18 [file medi-105-e49843-s018.docx]

## **Table S16.** Subgroup analysis of the total population

| **Variates** | **n**^†^ | **Events**^‡^ | **HR (95%CI)** | ***P* for interaction** |
| --- | --- | --- | --- | --- |
| **CKD risk stratification** | | | | < .000 |
| Non-CKD/Low risk | 34564 | 2643 | 1.020(1.015, 1.025) |  |
| Moderate risk | 8281 | 1901 | 1.020(1.014, 1.026) |  |
| High risk | 2782 | 1120 | 1.011(1.004, 1.018) |  |
| Very high risk | 1576 | 918 | 1.011(1.004, 1.018) |  |
| **CKD** | | | | .041 |
| No | 34564 | 2643 | 1.020(1.015, 1.025) |  |
| Yes | 12639 | 3939 | 1.016(1.012, 1.020) |  |

^†^ n refers to number of participants with different categories (unweighted)

^‡^ Event refers to number of all cause death events for participants with different categories (unweighted)

Subgroup analysis conducted on CKD (no/yes) and CKD risk stratification within total population in 1999-2018.

Model adjusted for Age, Gender, Race, Marital, Education levels, Body mass index, Smoking status, Serum Cotinine, Diabetes mellitus, Hypertension, Cardiovascular disease, Hyperlipidemia. But the model did not adjust for the stratification variables themselves.

Abbreviation: HR, hazard ratios; CI, confidence intervals; CKD, chronic kidney disease.
