# Supplementary material for: Tooth loss elevates all-cause and cause-specific mortality in adults with chronic kidney disease: The mediating role of frailty
Source: Medicine (Baltimore). 2026 Jul 24;105(30):e49843. doi: 10.1097/MD.0000000000049843 (PMC13406305; doi:10.1097/MD.0000000000049843)
Supplement: Supplementary file 20 [file medi-105-e49843-s020.docx]

## **Table S17.** HR (95% CIs) from sequential Cox models of tooth loss and all-cause mortality (per tooth and per 10 teeth)

|  | **HR (95%CI)** | **log(HR)** | **HR per 10 (95%CI)** | **log(HR per 10)** | ***P* value** |
| --- | --- | --- | --- | --- | --- |
| **Model 1**^†^ | 1.017(1.013, 1.021) | 0.017 | 1.183(1.135, 1.234) | 0.168 | < .000 |
| **Model 2**^‡^ | 1.013(1.009, 1.018) | 0.013 | 1.139(1.090, 1.190) | 0.130 | < .000 |
| **Model 3**^§^ | 1.012(1.008, 1.017) | 0.012 | 1.130(1.083, 1.179) | 0.122 | < .000 |

^†^ Model 1: Model adjusted for Age, Gender, Race, Marital, Education levels, Body mass index, Smoking status, Serum Cotinine, Diabetes mellitus, Hypertension, Cardiovascular disease, Hyperlipidemia

^‡^ Model 2: Model 1+ FI

^§^ Model 3: Model 2+ ln(hs-CRP)

^*^ HR per 10 refers to HR per unit of 10 teeth

Abbreviation: HR, hazard ratios; CI, confidence intervals.
