# Supplementary material for: Tooth loss elevates all-cause and cause-specific mortality in adults with chronic kidney disease: The mediating role of frailty
Source: Medicine (Baltimore). 2026 Jul 24;105(30):e49843. doi: 10.1097/MD.0000000000049843 (PMC13406305; doi:10.1097/MD.0000000000049843)
Supplement: Supplementary file 21 [file medi-105-e49843-s021.docx]

## **Table S18.** Pairwise differences in log hazard ratios and percentage attenuation between sequential models (per tooth and per 10 teeth)

| **Comparison** | **Δlog(HR)** ^a^ | **% attenuation vs. Model 1**^b^ | **Δlog(HR per 10)** | **% attenuation vs. Model 1** |
| --- | --- | --- | --- | --- |
| Model 2^‡^ − Model 1^†^ | -0.0037 | 22.34 | -0.0374 | 22.28 |
| Model 3 − Model 2 | -0.0008 | 4.71 | -0.0080 | 4.78 |
| Model 3^§^ − Model 1 (cumulative) | -0.0045 | 27.05 | -0.0454 | 27.06 |

^†^ Model 1: Model adjusted for Age, Gender, Race, Marital, Education levels, Body mass index, Smoking status, Serum Cotinine, Diabetes mellitus, Hypertension, Cardiovascular disease, Hyperlipidemia

^‡^ Model 2: Model 1+ FI

^§^ Model 3: Model 2+ ln(hs-CRP)

^a^ Δlog(HR) = log(HR_new) − log(HR_reference). Negative values indicate attenuation.

^b^ % attenuation vs. Model 1 = (logHR_M1 − logHR_Mk) / logHR_M1 × 100%.

Abbreviation: HR, hazard ratios; CI, confidence intervals.
