# Supplementary material for: Tooth loss elevates all-cause and cause-specific mortality in adults with chronic kidney disease: The mediating role of frailty
Source: Medicine (Baltimore). 2026 Jul 24;105(30):e49843. doi: 10.1097/MD.0000000000049843 (PMC13406305; doi:10.1097/MD.0000000000049843)
Supplement: Supplementary file 23 [file medi-105-e49843-s023.pptx]

## Slide 1
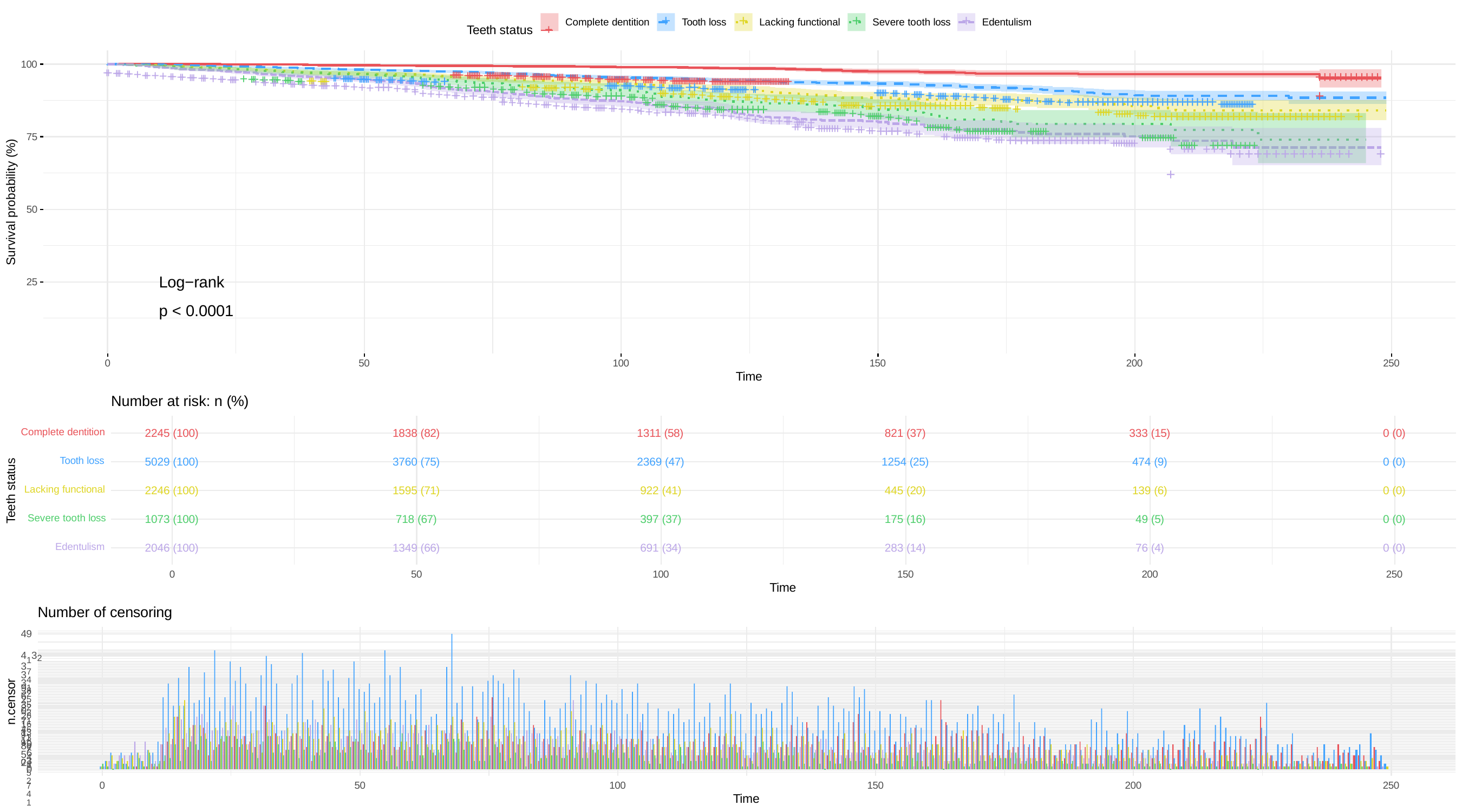

+
+
+
+
Teeth status +
Complete dentition
Tooth loss
Lacking functional
Severe tooth loss
Edentulism
100
++++++++++++++++++++++++++++++++++++++++++++++++++++++++++++++++++++++++++++++++++++++++++++++++++++++++++++++++++++++++++++++++++++++++++++++++++++++++++++++++++++++++++++++++++++++++++++++++++++++++++++++++++++++++++++++++++++++++++++
+++++++++++++
+++++++++++++++++++++++++++++++++++++++++++++++++++++++++++++++++++++++++++++++++++++++++++++++++++++++++++++++++++++++++
+++++++++++++++++++++++++++++++++++++++++++++++++++++++++++++++++++++++++++++++++++
++++++++++++++++++++++++++++++++++++++++++++++++++++++++++++++++++++++++++++++++++++++++++++++++++++
++++++++++++++++++++++++++++++++++++++++++++++++++
++++++++++++++++++++++++++++++++++++++++++++++++++++++++
++++++++++++++++++++++++++++++++++++++++++++++++++++++++
+++++++++++++++++++++++++++++++++++++++++++++++++ +
+++++++++++++++++++++++++++++++++++++++++++++++++++++++++ +++++++
+++++++++++++++++++++++++++++++++++++++++++++++++++++++++++++++ ++++++++++
75
+ +++ +++++++++++++++++	++
Survival probability (%)
50
Log−rank
25
p < 0.0001
0
50
100
150
200
250
Time
Number at risk: n (%)
Complete dentition
2245 (100)
1838 (82)
1311 (58)
821 (37)
333 (15)
0 (0)
Tooth loss
5029 (100)
3760 (75)
2369 (47)
1254 (25)
474 (9)
0 (0)
Teeth status
Lacking functional
2246 (100)
1595 (71)
922 (41)
445 (20)
139 (6)
0 (0)
Severe tooth loss
1073 (100)
718 (67)
397 (37)
175 (16)
49 (5)
0 (0)
Edentulism
2046 (100)
1349 (66)
691 (34)
283 (14)
76 (4)
0 (0)
0
50
100
150
200
250
Time
Number of censoring
49
4132
3798
3465
3132
n.censor
32089
2576
2243
21910
1687
1354
1021
987
654
321
0
0
50
100
150
200
250
Time
